# Dendritic Cell-Based Immunotherapy in Advanced Sarcoma and Neuroblastoma Pediatric Patients: Anti-cancer Treatment Preceding Monocyte Harvest Impairs the Immunostimulatory and Antigen-Presenting Behavior of DCs and Manufacturing Process Outcome

**DOI:** 10.3389/fonc.2019.01034

**Published:** 2019-10-25

**Authors:** Eva Hlavackova, Katerina Pilatova, Dasa Cerna, Iveta Selingerova, Peter Mudry, Pavel Mazanek, Lenka Fedorova, Jana Merhautova, Lucie Jureckova, Lukas Semerad, Rita Pacasova, Lucie Flajsarova, Lenka Souckova, Regina Demlova, Jaroslav Sterba, Dalibor Valik, Lenka Zdrazilova-Dubska

**Affiliations:** ^1^Department of Pharmacology, Faculty of Medicine, Masaryk University, Brno, Czechia; ^2^Department of Pediatric Oncology, University Hospital and Faculty of Medicine, Masaryk University, Brno, Czechia; ^3^Regional Centre for Applied Molecular Oncology, Masaryk Memorial Cancer Institute, Brno, Czechia; ^4^Department of Internal Medicine-Hematology and Oncology, University Hospital and Medical Faculty, Masaryk University, Brno, Czechia; ^5^Transfusion and Tissue Department, University Hospital Brno, Brno, Czechia

**Keywords:** dendritic cells, anti-cancer medications, sarcoma, neuroblastoma, cell-based medicinal products, investigator-initiated clinical trial, manufacturing outcome variability

## Abstract

Despite efforts to develop novel treatment strategies, refractory and relapsing sarcoma, and high-risk neuroblastoma continue to have poor prognoses and limited overall survival. Monocyte-derived dendritic cell (DC)-based anti-cancer immunotherapy represents a promising treatment modality in these neoplasias. A DC-based anti-cancer vaccine was evaluated for safety in an academic phase-I/II clinical trial for children, adolescents, and young adults with progressive, recurrent, or primarily metastatic high-risk tumors, mainly sarcomas and neuroblastomas. The DC vaccine was loaded with self-tumor antigens obtained from patient tumor tissue. DC vaccine quality was assessed in terms of DC yield, viability, immunophenotype, production of IL-12 and IL-10, and stimulation of allogenic donor T-cells and autologous T-cells in allo-MLR and auto-MLR, respectively. Here, we show that the outcome of the manufacture of DC-based vaccine is highly variable in terms of both DC yield and DC immunostimulatory properties. In 30% of cases, manufacturing resulted in a product that failed to meet medicinal product specifications and therefore was not released for administration to a patient. Focusing on the isolation of monocytes and the pharmacotherapy preceding monocyte harvest, we show that isolation of monocytes by elutriation is not superior to adherence on plastic in terms of DC yield, viability, or immunostimulatory capacity. Trial patients having undergone monocyte-interfering pharmacotherapy prior to monocyte harvest was associated with an impaired DC-based immunotherapy product outcome. Certain combinations of anti-cancer treatment resulted in a similar pattern of inadequate DC parameters, namely, a combination of temozolomide with irinotecan was associated with DCs showing poor maturation and decreased immunostimulatory features, and a combination of pazopanib, topotecan, and MTD-based cyclophosphamide was associated with poor monocyte differentiation and decreased DC immunostimulatory parameters. Searching for a surrogate marker predicting an adverse outcome of DC manufacture in the peripheral blood complete blood count prior to monocyte harvest, we observed an association between an increased number of immature granulocytes in peripheral blood and decreased potency of the DC-based product as quantified by allo-MLR. We conclude that the DC-manufacturing yield and the immunostimulatory quality of anti-cancer DC-based vaccines generated from the monocytes of patients were not influenced by the monocyte isolation modality but were detrimentally affected by the specific combination of anti-cancer agents used prior to monocyte harvest.

## Introduction

Several progressive and relapsing malignancies in pediatric patients have dismal life prognosis. Refractory neuroblastoma and refractory or metastatic sarcoma have an especially poor prognosis, with no consistently curative treatments available. Oberlin et al. (1) published a meta-analysis of North American and European studies on primary metastatic sarcomas and well-defined risk factors that—where two or more are present at presentation—distribute patients into a subgroup with only a 14% event-free and overall survival probability at 3 years from diagnosis. Patients over 10 years of age with limb primary or “other site” primary tumors with the alveolar subtype of rhabdomyosarcoma, bone marrow or bone involvements, and more than three metastatic sites are defined as having markers for a worse prognosis (1). Similar results were published in a study of relapsed rhabdomyosarsomas, with the prognosis for survival being < 10% at 5 years (2). In high-risk neuroblastoma, survival after relapse is poor, and the usual life expectancy is < 6 months. Based on our experience, patients with neuroblastomas with a high MIBG score after induction therapy have very poor 2-year survival (3). High-risk rhabdomyosarcomas are treated according to several globally accepted protocols with a combination of chemotherapy, surgery, and radiotherapy. Chemotherapy regimens consist of the alkylating agent ifosfamide or cyclophosphamide and vinca alkaloids combined with either etoposide or doxorubicin and actinomycin D. The cytotoxic chemotherapy regimens for relapsed and refractory neuroblastoma typically use a combination of camptothecins, topotecan, and irinotecan with agents such as cyclophosphamide and temozolomide, and achieve objective tumor responses but poor long-term outcomes. For such poor-prognosis patients, treatments with innovative and metronomic therapies (e.g., COMBAT, METRO) (4, 5), cell-based immunotherapies (6, 7), and novel molecularly targeted agents (8) are justified and are also effective in many cases, although their long-term effect has yet to be demonstrated.

DCs are essential antigen-presenting cells for the initiation, maintenance, and regulation of immune response (9). Active cancer immunotherapy directs the immune system to attack tumor cells by targeting tumor-associated antigens. We manufacture a fully personalized monocyte-derived dendritic cell-based vaccine that was evaluated in the investigator-initiated clinical trial “Combined antitumor therapy with *ex vivo* manipulated dendritic cells producing interleukin-12 in children, adolescents, and young adults with progressive, recurrent, or primarily metastatic high-risk tumors” (EudraCT number 2014-003388-39). The primary endpoint of the trial was an assessment of safety by analysis of the frequency of occurrence of AESI (adverse events of special interest). Vaccines that meet quality control (QC) requirements are registered for use and applied intradermally every 2–4 weeks for up to 35 doses.

Dendritic cell-based medical products are mostly manufactured through derivation from monocytes. Autologous monocytes are readily accessible and can be obtained from peripheral blood in sufficient amounts to prepare 10^7^-10^8^ DCs. Monocytes arise from hematological precursors in bone marrow, with a maturation time of 50–60 h (10), and enter the bloodstream for several days until their recruitment into tissues, where they possess the property to mature into tissue macrophages (11). Specifically, the classical CD14++ CD16– subpopulation representing 80–95% of circulating monocytes has a 1-day lifespan in circulation, the intermediate CD14+ CD16+ subpopulation (2–8% of circulating monocytes) has a 4-day lifespan, and the non-classical CD14+ CD16++ subpopulation (2–11% of circulating monocytes) has a 7-day lifespan in circulation (12–14). Monocyte count and function are influenced by various anti-cancer agents. Nevertheless, the published data on the impact of particular anti-cancer agents on the development and function of monocytes are scarce in comparison with those on hematologic toxicity toward neutrophils and lymphocytes. As most anti-cancer agents target DNA, they interfere with dividing cells including hematopoetic cells. Also, tyrosine kinase inhibitors (regorafenib, sunitinib, sorafenib) are associated with adverse events including hematological toxicities (15). Regorafenib hematological toxicity has been explained by the TK inhibition of FMS like tyrosine kinase 3 (FLT-3) and stem cell factor (c-KIT ligand), which represent hematopoietic growth receptors (15, 16). Reduction in the circulating monocyte count after sunitinib has been shown (17). Monocytes are also highly sensitive to the methylating agent temozolomide (TMZ) (18, 19). Cisplatin and carboplatin have been shown to alter monocyte differentiation to favor the generation of IL-10-producing M2 macrophages (20).

Various chemotherapeutics affect cell differentiation and the antigen presentation of DCs when treated *in vitro* during the differentiation process (21). Data are lacking on the potential *in vivo* impact of hematotoxic agents on the properties of medicinal products from monocyte-derived DCs. During the manufacture of DC-based anti-cancer immunotherapy under stringent GMP-compliant conditions, we experienced highly variable final product parameters in terms of both DC yield and immunostimulatory properties, and we hypothesized that hematotoxic anti-cancer therapy preceding monocyte harvest may influence the quality of DC-based medicinal products. The issue of the effect of pharmacotherapy on the quality of human monocyte-derived DCs cannot be reliably assessed in mimicked conditions by *in vitro* pretreatment of monocytes by anti-cancer agents. Thus, data addressing this issue can only be gathered retrospectively from real-life clinical conditions, such as our clinical trial, though with a limited number of patients included. Here, the Phase-I/II clinical trial protocol designed for heavily pre-treated cancer patients with heterogenic anti-cancer therapeutic protocols allows us to observe and analyze the effect of pharmacotherapy on the quality and presumably also on the anti-cancer action of *ex vivo*-manufactured DCs.

Therefore, our primary aims were to analyze the impact of (i) cytotoxic and targeted anti-cancer therapy preceding monocyte harvest and (ii) variability in the complete blood count on the quality of DC-based anti-cancer immunotherapy in high-risk sarcoma and neuroblastoma patients, representing the two main diagnoses in the DC clinical trial. A secondary aim was to reveal whether monocyte isolation by elutriation is superior to the isolation of monocytes through their adherence to plastic cultivation flasks.

## Methods

### Patients and Clinical Trial

#### Clinical Trial Eligibility and Allowed Medication

Patient eligibility/inclusion criteria for the clinical trial included being 1–25 years old male/female with histologically confirmed refractory, relapsing, or primarily metastatic high-risk tumors and having a performance status (Karnofsky or Lansky score) ≥ 50 and a life expectancy of longer than 10 weeks. Patients had to be clinically eligible for the surgical procedure to harvest tumor tissue for histological verification and tumor antigen extraction. Female patients had to have had a negative pregnancy test. All patients had to have adequate bone marrow, kidney, liver, and heart function, defined as absolute neutrophil count (ANC) ≥ 0.75 × 10^9^/L, thrombocytes ≥ 75 × 10^9^/L, hemoglobin 80 g/L, estimated glomerular filtration rate (eGFR) ≥ 70 mL/min/1.73 m^2^, serum creatinine ≤ 1.5-fold the upper limit for the appropriate age, bilirubin ≤ 1.5-fold the upper limit for the appropriate age, AST and ALT ≤ 2.5-fold the upper limit for the appropriate age, ejection fraction ≥ 50%, and fractional shortening ≥ 27% as assessed by echocardiography. In the case of bone marrow infiltration, the allowable ANC was ≥ 0.5 × 10^9^/L and blood platelets 40 × 10^9^/L. In case of liver metastases, AST and ALT had to be ≤ 5-fold the upper limit for the appropriate age. The exclusion criteria were as follows: seropositivity to HIV1,2, *Treponema pallidum*, hepatitis B or C, known hypersensitivity to the study medication, autoimmune disease that was not adequately treated, uncontrolled psychiatric disease, or uncontrolled hypertension defined as systolic and diastolic blood pressure over the 95th percentile for the appropriate age and height (patients ≤ 17 years old) or ≥ 160/90 mmHg or diastolic blood pressure ≥ 90 mmHg (patients ≥ 17 years old). Patients previously treated with dendritic cells or participating in another clinical trial during the 30 days before enrollment were not eligible to enter this clinical trial.

The allowed medication prior to monocyte harvest (leukapheresis) was as follows: metronomic chemotherapy, immune checkpoint inhibitors, and anti-CD20 antibodies were allowed as concomitant medication for any time before leukapheresis. Monoclonal antibodies (except anti-CD20), high-dose chemotherapy, and high-dose corticoids had to have been withdrawn at least 3 weeks prior to leukapheresis with the exception of corticoid treatment of brain edema, which was allowed. Since November 2017, an amendment has been made to the procedure for monocyte harvest, and tyrosine kinase inhibitors have to be withdrawn according to their half-life: drugs with a short half-life of 3–14 h must be withdrawn at least 2 days before leukapheresis (axitinib, dabrafenib, dasatinib, ibrutinib, idelalisib, nintedanib, ruxolitinib, and trametinib), drugs with a medium half-life of 15–35 h at least 7 days before leukapheresis (alectinib, bosutinib, lapatinib, lenvatinib, nilotinib, osimertinib, pazopanib, ponatinib, regorafenib, and non-TKI everolimus), and drugs with a long half-life of 36–60 h at least 12 days before leukapheresis (afatinib, ceritinib, erlotinib, gefitinib, imatinib, cabozantinib, crizotinib, sorafenib, sunitinib, vemurafenib, and non-TKI temsirolimus). Myelopoietic growth factors have to be withdrawn at least 7 days before leukapheresis/monocyte harvest.

#### Evaluation of Preceding and Concomitant Therapy

A precise analysis was performed of preceding and/or concomitant therapy 60 days before monocyte harvest for clinical trial subjects with neuroblastoma and sarcoma diagnoses. Data were mined from the clinical trial electronic case report form and the subjects' medical records. We particularly focused on therapeutic agents with a potential impact on the generation of DCs from monocytes and on DC immunostimulatory properties. These agents and the reports on their role in monocyte biology are summarized in Supplementary Table 1.

### DC Manufacture and Quality Control

Dendritic cell vaccine manufacture encompassed two phases—(i) preparation of tumor lysate as a source of the patient's tumor antigens and (ii) preparation of monocyte-derived DCs and their loading with tumor lysate. Quality control tests evaluated safety (negativity for pathogens), identity (cell immunophenotype), viability, and functions (cytokine production, stimulation of T-cells). The flow and decision tree of the manufacturing process is shown in Supplementary Figure 1.

#### Self-Tumor Antigen Extraction

Tumor lysate was prepared from the tumor tissue obtained from the patient during curative surgery or extended biopsy. In Clean Rooms, necrotic areas and connective tissue were removed from the tumor tissue with a surgical scalpel, keeping the specimen immersed in buffered solution. The remaining tissue was sliced into fragments of about 0.5 mm with a scalpel and forceps and then further crushed with the back of a syringe. Each suspension of tumor fragments and cells in HBSS was lysed through repeated (5 times) freezing in liquid nitrogen and thawing at 37°C. The crude tumor lysate was centrifuged at 450 g/7 min/4°C to remove particulate components. The tumor lysate was released for DC manufacture if the following criteria were met: (i) presence of viable tumor cells reported by a histopathologist, (ii) protein concentration, and (iii) microbiological sterility.

#### Peripheral Mononuclear Cell Collection

Monocytes were harvested as part of the mononuclear white blood cell (WBC) fraction. Mononuclear cells were collected from the peripheral blood of the patient using the Terumo BCT Spectra Optia Apheresis System. For collection, we used either an intermittent or continuous leukapheresis system. Due to its superior collection efficacy and easier procedure settings, we have preferred the continuous leukapheresis system since April 2018. A citrate dextrose solution, solution A (ACD-A), was used as an anticoagulant. In patients with a body weight of < 20 kg, anticoagulation with heparin was used to prevent citrate toxicity. The requirement for the minimal WBC count was 3 × 10^9^/L before the initiation of leukapheresis. To prevent risk of bleeding or ischemic complications during and after the procedure, hemoglobin of at least 80 g/L and platelets of at least 30 × 10^9^/L were required. In case of a patient with a body weight of < 20 kg, the leukapheresis set was pre-filled with donor erythrocytes. The aim of the leukapheresis was to obtain 60–80 mL of concentrate of mononuclear cells with a content of at least 0.5 × 10^9^ monocytes. Subsequent addition of 5% human albumin to the minimum required volume of 80 mL for further processing was allowed.

#### DC Manufacture in Clean Rooms

The numbers of WBCs, B-cells and T-cells, monocytes, and granulocytes in the leukapheretic product were evaluated using a hematology analyzer (XT-4000i, Sysmex) and flow cytometer (FC-500, Beckman Coulter) with staining for CD3 (clone UCHT1, Beckman Coulter) and CD19 (clone J3-119, Beckman Coulter). Monocytes for DC manufacture were separated from the leukapheresis product by either elutriation or adherence to a plastic surface. During elutriation (using an Elutra cell separator, Gambro BCT), blood cells were separated on the basis of sedimentation velocity into six fractions, where the last fraction rich in monocytes was used for DC manufacture. Contaminating cells after elutriation were mainly granulocytes with similar sedimentation velocity to monocytes. Five hundred million monocytes adhered for 2–4 h in three 175-cm^2^ tissue culture flasks with 35 mL of CellGenix® GMP DC Medium at 37°C/5% CO_2_ and were then washed with HBSS and processed further. Monocytes seeded from the elutriation product or attached by plastic adherence were then cultivated in three 175-cm^2^ tissue culture flasks with 70 mL of CellGenix® GMP DC medium supplemented with GM-CSF (1000 U/mL, CellGenix®) and IL-4 (320 U/mL, CellGenix®) at 37°C/5% CO_2_/6 days. On day 3, a fresh 70 mL of medium supplemented with the same concentration of GM-CSF and IL-4 was added to the culture. On day 6, immature DCs were exposed to autologous tumor lysate antigens (10 μg/mL) with added keyhole limpet haemocyanin (KLH, 1 μg/mL), IL-4 (320 U/mL), and GM-CSF (1000 U/mL) at 37°C/5% CO_2_/for 1.5–2 h. Maturation was induced by lipopolysaccharide (200 U/mL) and interferon-γ (50 ng/mL) for an additional 6 h at 37°C/5% CO_2_. Finally, cells were collected using accutase (Accutase®, Corning), counted in a Bürker cell chamber and frozen in aliquots of 2 × 10^6^ DCs in 100 μL of freezing medium CryoStor® CS2 at -80°C. All doses of the DC-based investigational medical product (IMP) named “MyDendrix®” were stored at -150°C until administration to the patient.

#### Quality Control of DC-Based Investigational Medicinal Product

DC characteristics were evaluated as a part of the quality control process of IMP from an aliquot of manufactured DC from each batch. The cryotube with DC was removed from a deep freezing box (-150°C) into a laminar flow box, quickly and gently thawed in hand while avoiding shaking, 1 mL of cold (2–8°C) DC medium (CellGenix® GMP-grade) was slowly added to the thawed DCs, and the DC suspension was transferred into 2 mL of cold DC medium. The DC suspension was handled at room temperature and processed immediately. DCs (8 × 10^5^ cells) were seeded into 1 well of a 6-well culture plate for sensitive adherent cells (Sarstedt, TC Plate 6-well, Cell+, growth area 8.87 cm^2^) and cultured in 3 mL of DC medium for 2 days (37°C/5% CO_2_) to obtain (i) medium containing cytokines produced by DCs during cultivation and (ii) mature DCs for phenotypic evaluation after 2 days of post-thaw cultivation. A 0.5 mL volume of medium containing DC-produced cytokines was collected after 23–25 h upon DC seeding and was centrifuged (10 min/410 g/4°C), and the supernatant was stored at -25°C for no longer than 30 days prior to analysis. For immunophenotypic evaluation of mature DCs, both detached and adherent DCs were harvested 47–49 h after DC seeding. The culture medium was collected and pooled with DCs harvested by accutase (0.5 mL/well 8.87 cm^2^/37°C) and centrifuged (5 min/410 g/20°C). The pellet was resuspended in 800 μL HBSS with 0.25% human albumin (Grifols) and processed immediately for immunophenotypic evaluation. Viability quantification was performed by propidium iodide (PI) exclusion assay. Briefly, 10^5^ DCs were stained with 10 μL of 1% PI in HBSS followed by immediate flow cytometric (Cytomics FC500) analysis of PI-positive events (= non-viable cells). The immunophenotype of DCs was evaluated in post-thaw DCs and in post-cultivation mature DCs. For the detection of each surface molecule, 0.5 × 10^5^ DCs were incubated for 20 min in the dark with the following antibodies: CD80-PC7 (clone MAB104, 10 μL), CD83-FITC (clone HB15e, 10 μL), CD86-PE (clone HA5.2B7, 10 μL), CD197-PE (clone G043H7, 10 μL), HLA-DR-PC5 (clone Immu357, 10 μL), CD14-PE (clone RMO52, 10 μL), or isotype controls IgG-PC5 (clone 679.1Mc7, 10 μL), IgG-PC7 (clone 679.1Mc7, 10 μL), IgG2a-FITC (clone 7T4-1F5, 10 μL), or IgG2a-PE (7T4-1F5, 10 μL), all from Beckman Coulter. Flow cytometric analysis was performed using a Cytomics FC500 with CXP software by manual gating on individual parameters, and the discrimination by appropriate isotype control was used to gate and quantify positive events. The concentrations of IL-12 and IL-10 in the DC culture medium were measured by flow cytometric bead assay (BD Biosciences) using internal quality controls (Quantikine® Immunoassay Control Group 1, R&D Systems). Absolute production of IL-12 or IL-10 per 10^6^ DC and the IL-12/IL-10 ratio were calculated. The allogenic (allo) and autologous (auto) stimulatory properties of DCs were examined by mixed lymphocyte reaction (MLR). In allo-MLR, the target cells were the peripheral blood mononuclear cells (PBMCs) obtained from pooled buffy coats from healthy donors. In auto-MLR, the target cells were the patient's lymphocytes separated by centrifugation in a density gradient using Histopaque-1077 (SigmaAldrich, density 1,077 g/mL) from the leukapheresis product obtained for DC manufacture. These pre-vaccination lymphocytes were cryopreserved using CryoStor CS5 medium (BioLife solutions) at -150°C and thawed prior to auto-MLR seeding. A sample of 10^7^ target lymphocytes were stained with 250 μL 10 μM carboxyfluorescein succidimidyl ester (CFSE, SigmaAldrich) and seeded into a sterile 96-well culture plate (Sarstedt, TC Plate 96-well, Suspension, F) at 10^5^ cells/well in 200 μL of complete X-vivo 10 medium (Lonza) containing 5% inactivated human male AB serum (SigmaAldrich) for the following: (i) 10^4^ DC/well in 10:1 target:effector MLR, (ii) positive control (PC) with phytohemagglutinin (PHA, SigmaAldrich) at a final concentration of 10 μg/mL, or (iii) negative control (NC) with complete X-vivo medium only. MLR experiments were seeded in triplicate and cultured for 6 days at 37°C/5% CO_2_. 2 × 10^4^ cells from each well were stained with CD3-PC7 (clone UCHT1, 10 μL/test, Beckmann Coulter) for flow cytometric detection of CFSE fluorescence on CD3+ T cells. Discrimination for dividing cells was set up using NC. T-cell proliferation was calculated as follows: [(average % of dividing T-cells in 10:1 MLR) – (average % of dividing T-cells in NC)] × 100/[(average % of dividing T-cells in PC) – (average % of dividing T-cells in NC)].

### Statistical Analysis

The Spearman correlation coefficient with a significance test was used to measure the strength of the relationship between patient CBC prior to leukapheresis, the parameters of the leukapheresis product, the DC yield, and the quality control parameters. Differences in parameter values between groups were assessed by the non-parametric Mann-Whitney or Kruskal-Wallis test. Hierarchical clustering analyses were performed using the complete linkage method with the distance based on the Spearman correlation coefficient. The Spearman correlation distance was used for clustering of batches, and the absolute Spearman correlation distance was used for clustering DC parameters. For clustering analyses, DC parameters were centered and scaled (Z-score of parameters). *P* < 0.05 were considered statistically significant. All statistical analyses were performed with R 3.5.3 software (22).

## Results

### Clinical Trial Accrual and Course

As of May 2019, 47 subjects were enrolled in the clinical trial, and the manufacturing process of DC-based vaccine was performed in 31 cases. Of these 31, the most common diagnoses were sarcoma, with 19 cases (61%), and high-risk neuroblastoma, with 4 cases (Table 1). In this group of 23 patients, we performed analysis of the manufacturing issues presented here. Sarcomas were specifically: seven Ewing sarcomas (36% of sarcoma pts), five (26%) osteosarcoma, two (11%) alveolar rhabdomyosarcoma, two (11%) embryonal rhabdomyosarcoma, and three (16%) synovial sarcoma (Table 1). The median enrollment age of the clinical trial was 14 years; 15 years for sarcoma patients and 5 years for neuroblastoma patients (Table 1). All 23 study subjects, i.e., 19 with sarcoma and four with neuroblastoma, underwent initial surgery to obtain tumor tissue for the tumor lysate-manufacturing process, and tumor lysates were manufactured without any tumor antigen extraction failure. Monocyte harvest and the subsequent manufacturing of DC-based IMP were performed for all 23 subjects. Out of the 23, 16 DC-based IMPs successfully passed through the manufacturing process and met the quality control criteria for administration to the patients. DC-based IMPs from seven subjects (six sarcoma, one neuroblastoma) were not manufactured or failed to pass quality control due to inadequate immunostimulatory properties (Table 1). The basic patient characteristics are described in Table 1, and the detailed clinical course is summarized in Supplementary Table 2.

**Table 1:** DC-based vaccine-manufacturing outcome, basic patient characteristics, therapy preceding monocyte harvest.

**Primary diagnosis**	**Date of study enrollment/Age in years at study enrollment/Pt No**	**Treatment line prior to monocyte harvest/Treatment and its duration/Date of monocyte harvest**	**DC-based vaccine-manufacturing outcome**
**EWING SARCOMA**			
Ewing sarcoma of the mandible	09/2015; 14; KDO-0101	2nd; VCR/Irino + pazopanib, 09/2015–04/2016; 01/2016	Passed QC
Localized Ewing sarcoma of the left femur	02/2016; 12; KDO-0109	3rd; ARST08P1 + sunitinib, 03/2016–06/2016; 03/2016	Did not pass QC
Localized Ewing sarcoma of the left distal humerus	02/2016; 12; KDO-0111	2nd; AEWS1031 + pazopanib, 02/2016–08/2016; 05/2016	Did not pass QC
Localized Ewing sarcoma of the spine C5-Th2, extradural, and intraspinal involvement	08/2016; 24; KDO-0118	2nd; AEWS1031, 08/2016–02/2017, 2 cycles VTC, 2 cycles VCR/Irino; 01/2017	Passed QC
Ewing sarcoma of the pelvis	12/2016; 14; KDO-0121	1st; Euro Ewing 2008, 11/2016–05/2017; 06/2017	Did not pass QC
Ewing sarcoma of the left proximal tibia	12/2016; 15; KDO-0122	2nd; VTC cycles, 01/2017–05/2017; 03/2017	Did not pass QC
Localized Ewing sarcoma of the left tibia	08/2018; 22; KDO-0144	2nd; 2x TMZ/Irino, 08/2018–10/2018; 10/2018	Did not pass QC
**OSTEOSARCOMA**			
Localized high-grade osteosarcoma of the right distal femur	09/2015; 10; KDO-0102	4th; VCR/Irino + pazopanib; 12/2015	Passed QC
High grade osteoblastic osteosarcoma of the left distal femur	10/2016; 8; KDO-0120	1st; AOST 0331, 10/2016–07/2017; 03/2017	Not manufactured
Localized osteoblastic osteosarcoma of the right proximal tibia	01/2017; 18; KDO-0124	3rd; AOST 1321 + VBL + CPM, 02/2017–10/2017; 3/2017	Passed QC
Localized osteosarcoma of the right proximal femur	02/2018; 25; KDO-0133	2nd; COMBAT III, 04/2018–12/2018; 04/2018	Passed QC
High-grade osteoblastic osteosarcoma of the left distal femur	05/2018; 22; KDO-0139	2nd; AOST0331 – cycle IE 07/2018; 09/2018	Passed QC
**ALVEOLAR RHABDOMYOSARCOMA**			
Alveolar rhabdomyosarcoma of the right calf	10/2015; 14; KDO-0103	2nd; ARST 0921 + TEM, 11/2015–01/2016; 12/2015	Passed QC
Alveolar rhabomyosarcoma, primum ignotum	10/2016; 12; KDO-0119	1st; ARST08P1 + TEM, 10/2016–05/2018; 04/2017	Passed QC
**EMBRYONAL RHABDOMYOSARCOMA**			
Embryonal rhabomyosarcoma of the pelvis	09/2017; 18; KDO-0131	1st; EpSSG RMS 2005, 09/2017–06/2018; 01/2017	Passed QC
Localized embryonal rhabomyosarcoma of the pelvis	07/2018; 15; KDO-0143	3rd; - rEECur - Topo/CYC, 08/2018–12/2018; 09/2018	Passed QC
**SYNOVIALOSARCOMA**			
Synovial sarcoma of the left thigh	04/2016; 14; KDO-0114	1st followed by COMBAT III 05/2015–12/2016; 12/2016	Passed QC
Localized synovial sarcoma of the neck	04/2018; 17; KDO-0137	2nd; Modified COMBAT III from 04/2018 + pazopanib from 08/2018; 06/2018	Passed QC
Localized synovial sarcoma of the left calf	06/2018; 21; KDO-0141	2nd; COMBAT III modified, 08/2018–02/2019; 10/2018	Passed QC
**NEUROBLASTOMA**			
Neuroblastoma in the retroperitoneum	04/2016; 12; KDO-0115	2nd; METRO-NB2012, 05/2016–10/2016; 07/2016	Passed QC
High-risk neuroblastoma in the left glandula suprarenalis	02/2018; 4; KDO-0135	1st followed by dinutuximab + retinoic acid, 11/2018–02/2019; 02/2019	Passed QC
Neuroblastoma in the right retroperitoneum	07/2018; 3; KDO-0142	2nd; ANBL 1221 - 3 cycles TMZ/Irino + dinutuximab, 08/2018–11/2018; 08/2018	Did not pass QC
Neuroblastoma in the right glandula suprarenalis	10/2018; 6; KDO-0147	4th; METRO-NB2012, 05/2017–12/2018; 11/2018	Passed QC

*CPM, cyclophosphamide; Irino, irinotecan; TEM, temsirolimus; TMZ, temozolomide; Topo, topotecan; VBL, vinblastine; VCR, vincristine; IE, ifosfamide etoposid; VTC, vincristine, topotecan, cyclophosphamide; Pt. No., patient number; QC, quality control. Chemotherapy protocols: AEWS1031 (Ewing sarcoma)—vincristine, doxorubcin, cyclophosphamide, ifosfamide, etoposide; AOST0331 (osteosarcoma)—cisplatin, doxorubicine, methotrexate; AOST1321 (osteosarcoma)—denosumab; ARST0921 (refractory or relapsed rhabdomyosarcoma)—bevacizumab, vinorelbine, cyclophosphamide and temsirolimus; ARST1321 (non-rhabdomyosarcoma soft tissue sarcomas)—ifosfamide, doxorubicin, pazopanib; COMBAT III (metronomic)—celecoxib, etoposide, temozolomide, fenofibrate, ergocalciferol, bevacizumab, vinorelbine, cis-retinoic acid; EpSSG RMS 2005 (rhabdomyosarcoma)—ifosfamide, vincristine, actinomycin, doxorubicin; Euro Ewing (Ewing sarcoma)—vincristine, ifosfamide, doxorubicin, etoposide, actinomycin, cyclophosphamide; METRO-NBL2012 (metronomic treatment for neuroblastoma)—etoposide, celecoxib, propranolol, cyclophosphamide, vinblastine; rEECur protocol (relapsed soft tissue sarcoma)—topotecan, cyclophosphamide, irinotecan, temozolomide. Details on anti-cancer therapy dosing are summarized in Supplementary Table 2*.

### Dendritic Cell Manufacturing, Its Yield, and DC Quality Including Immunostimulatory Properties

We achieved DC yields ranging from 0 to 43.6%, with a mean of 17.2% and an s.d. of 12.7% in this specific cohort. A DC yield equal to 0 represented a manufacturing process that was unsuccessful, with all DCs detached from the flasks. The quality control parameters involved microbial sterility and *Mycoplasma* spp. negativity, the viability and phenotype of thawed DCs, the phenotype of thawed DCs after 2-day cultivation, the production of IL-12 and IL-10 during 24-h cultivation of thawed DCs, and 6-day allo-MLR and auto-MLR. All batches of DCs fulfilled the microbiological criteria of QC and the criteria of viability, ranging from 85 to 100% with a mean of 95%. Their variability in phenotype and immunostimulatory property is shown in Supplementary Table 3. The mean phenotype of the manufactured DCs immediately after thawing for selected parameters was as follows: CD8019 (range: 2–86%), CD86 91% (76–100%), CD83 21% (0–86%), CD14 20% (1–69%), and CD197 90% (73–99%). The mean phenotype of thawed DCs after 2-day cultivation for selected parameters was as follows: CD80 77% (range: 25–97%), CD86 99% (95–100%), CD83 61% (12–89%), and MHC II 93% (63–100%). Mean cytokine production was as follows: IL-12 8,327 pg/10^6^ DC (range: 9–80,824 pg/10^6^ DC), IL-10 280 pg/10^6^ DC (6–1,731 pg/10^6^ DC), and IL-12/IL-10 ratio 35 (1–246). The mean *in vitro* proliferation of T-cells stimulated by manufactured DCs was 67% (29–98%) in allo-MLR and 9% (−3–37%) in auto-MLR. Due to inappropriate results for the immunostimulatory parameters of QC (phenotype, cytokine production, MLR), six out of 22 (27%) of the manufactured batches of DCs were not released for use in the clinical trial. The parameter values of the manufactured batches of DCs are shown in Supplementary Table 3.

#### Isolation of Monocytes by Adherence vs. Elutriation and Its Impact on Manufacturing Process Yield and the Immunostimulatory Parameters of DCs

Isolation of monocytes for DC manufacture was performed by elutriation in 14 cases and by plastic adherence in nine (39%) cases based on the real-world situation. Until March 2017, we performed elutriation of the leukapheresis product in all cases (11 cases: KDO-0101, -0102, -0103, -0109, -0111, -0114, -0115, -0118, -0120, -0122, -0124). Between April and September 2018, we performed elutriation in cases KDO-0121, -0137, and -0139, and adherence to plastic in cases KDO-0133, -0142, and -0144 due to there being > 10% neutrophils in the leukapheresis product or technical issues with the Elutra device for KDO-0119 and -0131. After October 2018, we isolated monocytes exclusively by adherence to the plastic surface in all cases: KDO-0135, -0141, -0144, and -0147.

**Figure 1:**
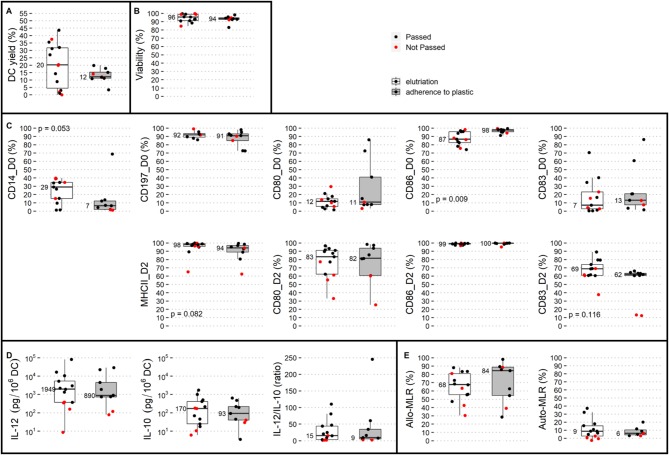
Comparison of two monocyte isolation modalities with respect to dendritic cell (DC) production. Elutriation (white box plots) and adherence to plastic (gray box plots) were compared based on QC parameters: **(A)** DC yield, and post-thaw: **(B)** viability, **(C)** DC phenotype on day 0: CD14, CD197, CD80, CD86, and CD83 and on day 2: MHC II, CD80, CD86, and CD83, and immunostimulatory properties presented by **(D)** IL-12 production, IL-10 production, and IL-12/IL-10 production ratio, **(E)** allo-MLR and auto-MLR. Median values are shown for each parameter for each monocyte isolation modality. Black dots show QC results of manufactured DCs that passed quality control, and red dots show results of manufactured DCs that did not pass quality control.

Addressing the issue of whether the elutriation process is superior to adherence to plastic retrospectively, we compared the proportions of batches passing QC and their DC yield and phenotypic and immunostimulatory properties under the two methods. Adherence to plastic resulted in two (22%) batches not being released, and elutriation resulted in five (36%) batches not being released (four did not pass QC and one was not manufactured). The OR (odds ratio) for passing QC in the plastic-adherence modality was 1.94 (95% CI: 0.29–13.19). The DC yield, viability, phenotype, and immunostimulatory properties (IL-12, IL-10, the IL-12/IL-10 ratio, allo-MLR, auto-MLR) in adherence to plastic vs. elutriation are summarized in Figure 1. A statistically significant difference was observed between QC results and monocyte isolation modality for the following post-thaw parameters (i) DC expression of CD86 on day 0 that was higher in the manufacturing process with plastic adherence, and (ii) borderline significant expression of CD14 on day 0 that was higher with elutriation. The values of both parameters were in favor of adherence to plastic. It is of note here that the subgroup with isolation of monocytes by the adherence to plastic was not biased by including a higher proportion of cases without potentially monocyte-interfering pharmacotherapy (“m” vs. “0” as described later; *p* = 0.643). Thus, we conclude that the isolation of monocytes by adherence to plastic is comparable to a manufacturing process with monocyte elutriation.

#### Parameters of CBC Prior to Monocyte Harvest, and Parameters of the Leukapheresis Product and Their Impact on Manufacturing Process Yield and the Immunostimulatory Properties of DCs

**Figure 2:**
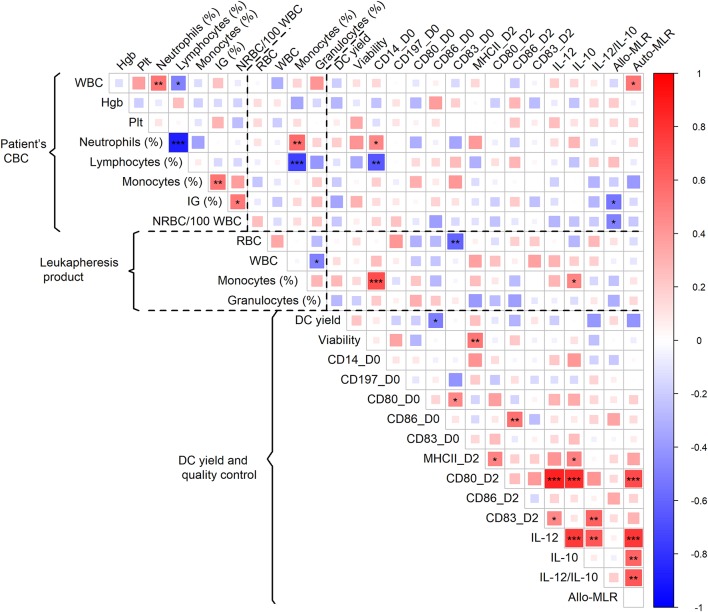
Association of patient CBC prior to monocyte harvest and parameters of leukapheresis product with DC yield and quality control. Red color represents a positive correlation and blue color a negative correlation; strength of relationship is represented by size of square and intensity of color—larger squares with intense color have a stronger association; ^*^p < 0.05, ^**^p < 0.01, ^***^p < 0.001.

With the aim of identifying the CBC parameters (shown for each batch in Supplementary Table 3) associated with adequate DC characteristics and thus predicting whether the DC-manufacturing process would pass QC, we analyzed CBC prior to monocyte harvest in the context of batches that fail to pass QC and DC yield, phenotype, and immunostimulatory properties. The presence of immature granulocytes in CBC was associated with unsuccessful manufacturing (*p* = 0.046). DC yield was not associated with any single parameter of CBC. Expression of CD14 on manufactured cells was negatively correlated with relative lymphocyte count in CBC (*p* = 0.001) (Figure 2). The level of allogenic MLR was negatively associated with both the presence of immature granulocytes (*p* = 0.010) and NRBC (*p* = 0.018) in pre-leukapheresis CBC (Figure 2). The level of autologous MLR was positively associated with absolute leukocyte count (*p* = 0.016) (Figure 2). Similarly, a high proportion of monocytes (*p* < 0.001) and low proportion of T-cells (*p* = 0.001) in the leukapheresis product were associated with increased expression of CD14 on manufactured cells (Figure 2). A high proportion of monocytes in the leukapheresis product was associated with increased production of IL-10 by manufactured cells (*p* = 0.027) (Figure 2).

#### Therapy Preceding and/or Concomitant With Monocyte Harvest and Its Association With Manufacturing Process Yield and the Immunostimulatory Properties of DCs

**Figure 3:**
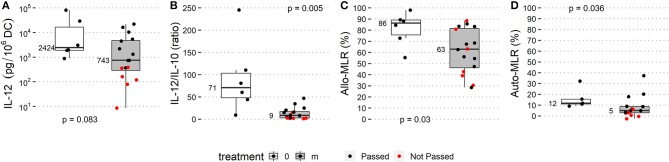
Treatment prior to monocyte harvest and immunostimulatory properties of manufactured DCs. Manufacturing subgroup from monocytes harvested after MTD-based therapy potentially interfering with monocyte biology (listed in Supplementary Table 1; “m” treatment, gray box plots) and manufacturing subgroup from monocytes from untreated patients or after non-interfering treatment (“0” treatment, white box plots) were compared based on QC parameters: **(A)** IL-12 production, **(B)** IL-12/IL-10 production ratio, **(C)** allo-MLR and **(D)** auto-MLR. Median values are shown for each parameter for each treatment subgroup. Black dots show QC results of manufactured DCs that passed quality control, and red dots show results of manufactured DCs that did not pass quality control.

The patient history of anti-cancer treatment and the outcome of DC manufacture were evaluated for an association between DC parameters and lines of therapy classified as 1st, 2nd, and 3rd or subsequent lines that were followed by monocyte harvest for DCs. The history of anti-cancer treatment had no observed impact on the quality of manufactured DCs (Supplementary Figure 2). Pharmacotherapeutics 60 days prior to and/or concomitant to monocyte harvest were classified into two groups and designated as follows (i) “m” (*n* = 17) for administration of therapy potentially interfering with monocyte viability and/or differentiation, namely TKI, mTOR inhibitors, chemotherapy in cell biology-interfering doses, i.e., MTD-based dose, anti-RANKL mAb, retinoic acid, and/or G-CSF (Supplementary Table 1) < 60 days prior to monocyte harvest, (ii) “0” (*n* = 6) for metronomic therapy/chemotherapy or no potentially monocyte-interfering therapy concomitantly or < 60 days prior to monocyte harvest. All batches from the “0” category passed QC, whereas seven out of 17 (41%) monocyte-derived DCs from the “m” category failed to be released for patient administration. The OR for passing QC in category “0” was 9.3 (95% CI: 0.5–191). DC yield, DC immunophenotype on day 0 and day 2, and production of IL-10 did not differ between the “0” and “m” categories (Supplementary Figure 3). Median IL-12 production was 2,424 pg/10^6^ DCs in the “0” category and 743 pg/10^6^ DCs in category “m” (*p* = 0.083). The median IL-12/IL-10 ratio was 71 in the “0” category and 9 in the “m” category (*p* = 0.002). The median T-cell proliferation in allo-MLR was 86% in the “0” category and 63% in the “m” category (*p* = 0.027), and the in auto-MLR was 12% in the category “0” and 5% in category “m” (*p* = 0.036) (Figure 3).

**Figure 4:**
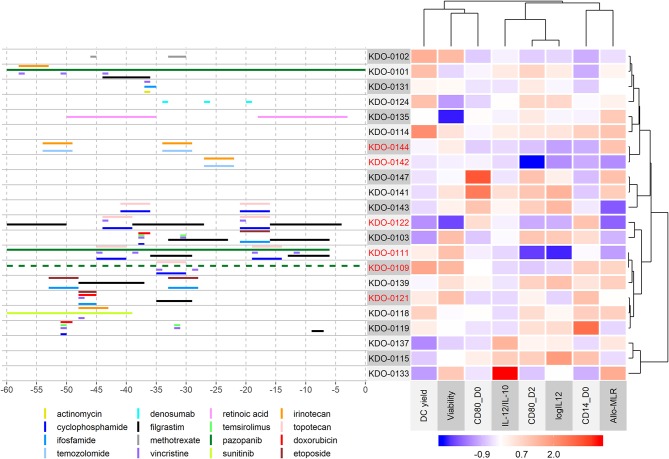
Cluster analysis of DC parameters in the context of therapy prior to monocyte harvest. The heatmap on the right shows the immunostimulatory properties of manufactured DCs centered and scaled in the column direction (Z-score of parameters). Clusters are based on correlations. For clustering of DC parameters, but not batches, an equal meaning to positive and negative correlations was considered, and therefore strongly correlated parameters in the positive or negative manner clustered together. The left panel shows the treatment administered within 60 days of monocyte harvest. The day of the mononuclear harvest was set as day 0. An interactive version of the left panel with a detailed description of treatment including dosing is provided in Supplementary Material 1. Metronomic doses of chemotherapeutic drugs and supportive therapy such as vitamins and probiotics are not shown here but are summarized in Supplementary Table 2. Batches that did not pass quality control are indicated in red.

In the analyzed study cohort, therapeutic regimens were heterogenic, with patients often treated with a combination of various compounds prior to monocyte harvest, and thus further categorization into single agent-defined subgroups and their analysis were impossible. Therefore, we performed cluster analysis of DC parameters in the context of therapy prior to monocyte harvest (Figure 4). Here we observed a cluster defined mainly by a superior IL-12/IL-10 ratio but low DC yield comprising batches KDO-0133 without any anti-cancer treatment, KDO-0137 treated with metronomic modified COMBAT with celecoxib, fenofibrate, low-dose cyclophosphamide, and low-dose vinblastine, and KDO-0115 treated with metronomic therapy with low-dose vinblastine, celecoxib, low-dose cyclophosphamide, and propranolol (see Supplementary Table 2 for details on the treatment schedule and dosing). Furthermore, we observed a very similar pattern in DC properties in two batches, KDO-0142 and KDO-0144, that were manufactured from monocytes obtained from patients treated with temozolomide and irinotecan. These batches exhibited robust monocyte differentiation, as represented by their low CD14 expression, but failed to produce IL-12 or an immunostimulatory phenotype when matured, as represented by CD80 on post-cultivation DCs on day 2, and therefore did not meet the QC criteria. A pattern of relatively low DC yield, high production of IL-12, and notable monocyte differentiation and DC immunostimulatory phenotype and function was observed for batches KDO-0147, generated from monocytes from patients treated with celecoxib, and KDO-0141, from patients pretreated with combined metronomic therapy with low-dose vinblastine, low-dose etoposide, celecoxib, cholecalciferol, and fenofibrate. Batches KDO-0103 and KDO-0122 similarly exhibited poor yield, poor monocyte differentiation, a rather low IL-12/IL-10 ratio, and very low immunostimulatory functions toward donor T-cells. Monocytes from both batches were pretreated with an MTD-based combination of topoisomerase inhibitor and alkylating agent, with last administration from day 21 to 17, namely etoposide and ifosfamide in KDO-0103 and topotecan and cyclophosphamide in KDO-0122. This was followed in both cases by 9 days of administration of G-CSF filgrastim up to 7 days prior to monocyte harvest. High DC yield and viability but low markers of differentiation, immunostimulatory phenotype and IL-12/IL-10 ratio were similarly observed for batches KDO-0111 and KDO-0109 treated with topotecan, cyclophosphamide, and pazopanib. Based on features such as good DC yield and viability but low monocyte differentiation and a below-average IL-12/IL-10 ratio, these two batches clustered with KDO-0139 (treated with etoposide, ifosfamide, and filgrastim), KDO-0121 (etoposide, ifosfamide, and filgrastim), KDO-0118 (irinotecan and sunitinib), and KDO-0119 (cyclophosphamide, temsirolimus, and filgrastim). Notably, monocytes affected by retinoic acid (KDO-0135) or anti-RANKL denosumab (KDO-0124) produced DCs of average quality. In summary, monocyte-interfering MTD-based treatment of the clinical trial patients prior to monocyte harvest was associated with an impaired DC-based immunotherapy manufacturing process outcome. Certain combinations of anti-cancer treatments elicited a similar pattern of inadequate DC parameters. Namely, a combination of temozolomide and irinotecan was associated with poor DC maturation and immunostimulatory features, and a combination of pazopanib, topotecan, and MTD-based cyclophosphamide was associated with poor DC differentiation maturation and immunostimulatory parameters.

## Discussion

Here we show that despite strict adherence to the validated manufacturing protocol, the outcome of the manufacture of the medicinal product with monocyte-derived DCs is highly variable in terms of both DC yield and immunostimulatory properties. Moreover, in 30% of cases, manufacture of DC-based immunotherapy for advanced sarcoma and high-risk neuroblastoma patients resulted in a product that did not meet the specifications for the medicinal product and therefore was not released for application. This product failure rate was higher than in published studies (23, 24). Thus, in an attempt to improve the manufacturing process, to predict DC-manufacturing outcome, and, subsequently, to avoid laborious and costly DC manufacture that would not meet QC specifications, we addressed key variables in the manufacturing process. Namely, we focused on the issues of (i) monocyte isolation from the mononuclear leukapheresis product, (ii) parameters of the patient's CBC prior to monocyte harvest and parameters of the leukapheresis product, and (iii) anti-cancer therapy preceding monocyte harvest that may interfere with the ability of monocytes to differentiate into immunostimulatory DCs.

Regarding the method of monocyte isolation, we assessed whether monocyte extraction by a simple method of adherence to a plastic surface is comparable to the elaborate method of elutriation. During elutriation, monocytes can be contaminated with granulocytes with a similar sedimentation velocity to monocytes. Based on this observation, we validated the DC-manufacturing process with isolation of monocytes by adherence to plastic (25) to avoid contaminants that may interfere with DC differentiation by altering the levels of pro-differentiation cytokines and/or the formation of a suppressing microenvironment through generating decay products during cultivation. By comparative analysis of DC yield and immunostimulatory properties from the manufacturing processes of isolation of monocytes by elutriation vs. adherence to plastic, we conclude that the adherence method is comparable to the elutriation method. The method of adherence to plastic is simple in terms of the equipment, material, and manufacturing steps required and therefore is less costly, less prone to errors, and more GMP-friendly than the elutriation process. In healthy adult volunteers, monocyte-derived DC yield with monocyte elutriation has been shown to be superior to adherence to plastic (26); this was not observed under our manufacturing conditions of heavily pretreated pediatric sarcoma and neuroblastoma patients.

With regards to the pharmacotherapy preceding monocyte harvest, we observed that therapy with agents interfering with the biology of monocytes 60 days prior to monocyte harvest was associated with reduced production of IL-12 and deficient functional immunostimulatory properties of the manufactured DC-based vaccine and subsequently often resulted in QC failure. It is of note here that failures in DC production occurred more often prior to the implementation of stricter criteria for non-allowed pharmacotherapy preceding monocyte harvest. Specifically, we observed impaired monocyte differentiation and, subsequently, inadequate immunostimulatory features in monocytes pretreated with a combination of an MTD-based dose of the alkylating agent cyclophosphamide, topoisomerase I inhibitor topotecan, and TKI pazopanib. We have previously shown that TKI pazopanib *in vitro* impairs the immunostimulatory properties of monocytes, including up-regulation of the immunoinhibitory surface molecule ILT-3 and decreased capability to up-regulate MHC II in response to LPS (27). Interestingly, however, pretreatment of monocytes *in vivo* with pazopanib without any other immediate treatment (KDO-0101) did not result in attenuated DC vaccine quality. Topotecan has been shown to partially activate monocyte-derived DCs but to prevent the full maturation of DCs stimulated with a cocktail of proinflammatory mediators (28). A different pattern was observed for DCs from cases treated with a combination of the alkylating agent temozolomide (TMZ) and the topoisomerase I inhibitor irinotecan (iri), and we observed monocyte differentiation but not DC immunostimulatory properties, resulting in a medicinal product that did not pass QC and was not administered. It is of note that one case was a sarcoma and one a neuroblastoma patient. Moreover, we also observed a similar pattern of poor DC parameters in a case of synovialosarcoma with TMZ/iri therapy in a cohort of patients outside this clinical trial. It has been shown that monocytes are particularly sensitive to the methylating agent temozolomide, undergoing apoptosis, while monocyte-derived DCs and macrophages are resistant to TMZ (19). Briegert and Kaina and Bauer et al. showed that monocytes accumulated single-strand DNA breaks due to failure of the re-ligation step in base excision repair and showed a lack of DNA repair protein expression (18, 19). Following TMZ treatment, monocytes demonstrated an unbalanced expression of DNA repair proteins, impairing base excision repair and the accumulation of double-stranded breaks (18, 19). *In vitro* studies of TMZ/iri cytotoxicity to neuroblastoma cells have revealed single- or double-stranded DNA damage to be mostly due to SN-38 (the active metabolite of irinotecan) and to be further enhanced through the addition of TMZ (29). Thus, we hypothesize that DNA damage caused by the combination of irinotecan and TMZ in the context of particular hypersensitivity of monocytes to temozolomide may underlie the unfavorable effect of anti-cancer therapy with TMZ/iri on the monocyte-derived immunostimulatory DC-manufacturing process. Monocytes from a patient treated with methotrexate, doxorubicin, and cisplatin failed to produce viable dendritic cells, but monocytes from another patient treated with methotrexate did not fail to produce DC vaccine. Methotrexate has reportedly inducedl apoptosis, reduced viability, induced differentiation, and reduced inflammatory properties of monocytes (30–33), and we may speculate, although based on anecdotal observation, that if combined with cisplatin, thereby shifting monocyte differentiation into an immunosuppressive phenotype (20), methotrexate may result in failure of monocyte-derived DC generation.

Regarding the composition of pre-leukapheresis CBC and the derived leukapheresis product and the outcome of DC manufacture, we observed that three interconnected features, i.e., (i) a low relative lymphocyte count, (ii) a high relative neutrophil count in CBC, and (iii) a high proportion of monocytes in the leukapheresis product, were associated with unfavorably high expression of CD14 on the manufactured cell product. Moreover, the presence of an increased number of immature granulocytes was associated with decreased potency of the DC-based product as quantified by allo-MLR. These observations may be underlain by emergency myelopoesis stimulated by G-CSF, which leads to a quantitative and qualitative change in all circulating myeloid cell types including neutrophils, monocytes, and myeloid-derived suppressor cells (34, 35). While fostering granulocyte effector functions, G-CSF also seems to promote immunosuppressive and tolerogenic properties in monocytes and monocyte-derived cells including increased production of IL-10 (36–39). In this context, it is of note that six out of seven cases treated with G-CSF within 60 days prior to monocyte harvest exhibited donor T-cell stimulation below the average and that the level of T-cell stimulation decreased with the intensity of G-CSF prior to monocyte harvest. Although the effect of G-CSF treatment on the DC-manufacturing process in our study cannot be dissected from the effect of preceding chemotherapy and targeted therapy, the tentative interpretation is that stimulation of myelopoesis with growth factors of granulocytes may have a rather negative impact on the outcome of the DC-based vaccine-manufacturing process.

Here, we show that treatment of patients with certain anti-cancer agents in MTD-based doses prior to monocyte harvest often leads to failure of manufacture of the immunostimulatory DC-based vaccine. We propose that the optimal time for monocyte harvest for generating DCs is prior to a cell-interfering treatment. With respect to the DC-manufacturing workflow, this would mean, in a majority of cancer patients, the implementation of DC manufacture from cryopreserved monocytes. Several studies have investigated the effect of cryopreservation on monocyte differentiation into DCs, but results have been conflicting. Some studies observed cryopreservation to have no effect on monocyte-derived DC production (40, 41). On the other hand, Silveira et al. showed that, when compared to fresh monocytes, cryopreserved monocytes exhibited impaired differentiation into dendritic cells, with lower rates of maturation and cytokine production in response to LPS and lower lymphocyte proliferation in allo-MLR (42). Thus, the cryopreservation of monocytes for DC generation may decrease the quality of manufactured DCs, and the level of this decrease needs to be specified for a particular manufacturing protocol. In case of a minor drop in DC maturation and immunostimulatory parameters and function due to the cryopreservation of monocytes, this manufacturing modality should be considered, as it would allow harvesting of therapy-naïve monocytes and avoid a potentially detrimental effect of certain anti-cancer and supportive treatment on the quality of DC-based anti-cancer immunotherapy.

Another issue in the context of the concurrence of anti-cancer treatment and monocyte-derived DC manufacture is the length of the pharmacotherapy-free period prior to monocyte harvest. From our real-life experience gained on this study group, we conclude that a 30-day interval without treatment is not sufficient for the combination of temozolomide and irinotecan to sufficiently wash out the monocyte biology-interfering effect of this combination. However, the issue of a safe therapy-free window is not likely to be addressable through the establishment of a wash-out period for a particular drug. The fitness of monocytes and their capacity to differentiate and mature into DCs with high antigen-presenting effect is a matter of their biological function in the context of iatrogenic affection, which is complexly shaped by the need for immediate treatments, their combinations, their cumulative doses, and the long-term history of treatment. Therefore, identifying a marker revealed from a patient's peripheral blood that predicts the outcome of DC-generation would help to avoid an unproductive anti-cancer DC-manufacturing process. Here we show that a high monocyte count in CBC is not predictive of an efficacious outcome for DC generation. Nevertheless, we find that the presence of immature granulocytes in CBC may predict decreased immunostimulation elicited by DCs and, subsequently, unsuccessful preparation of DC-based IMP. However, closer evaluation of monocyte function prior to their collection for DC generation may be considered. A surrogate marker for the immunostimulatory capacity of monocytes may be evaluated in (i) their phenotype, e.g., the level of HLA-DR or ILT-3 expression on monocytes or the proportion of particular monocyte subsets according to CD14 and CD16 expression, or (ii) their ability to produce pro-inflammatory cytokines upon TLR stimulation (27).

In summary, monocytes represent a key starting material for anti-cancer DC-based vaccine manufacture. Therefore, monocyte conditions have an impact on the manufacturing yield, the differentiation into DCs, and the level of maturation and subsequent immunostimulatory functions. For DC manufacture from heavily pretreated pediatric patients with high-risk sarcomas and neuroblastoma, we conclude that the manufacturing yield and immunostimulatory quality of anti-cancer DC-based vaccine generated from patient's monocytes were not influenced by the monocyte isolation modality but were detrimentally affected by certain combinations of anti-cancer agents. Thus, the combination of chemotherapy or targeted therapy with DC-based immunotherapy needs to be scheduled not only with respect to the likely beneficial role of anti-cancer agents on the immunogenicity of tumor antigens for both *in vitro* DC generation via induction of immunogenic cell death and *in vivo* for effector response of DC-activated T-cells but also with respect to optimal monocyte immunostimulatory functions. Finally, these findings may also have implications for the general pharmacology of anticancer treatment. As our model of *ex vivo*-activated DC preparation generally parallels the *in vivo* differentiation pathways of monocytes to the antigen-presenting cells, we may imply that drug combinations at doses used clinically may result in an impairment of patient DCs and possibly immune competence in general. In conclusion, these findings may stimulate further research on dose and mechanism-of-action-based drug combination in patient-centered trials to optimize the treatment modalities currently available in clinical oncology.

## Data Availability Statement

All datasets generated for this study are included in the manuscript/Supplementary Files.

## Ethics Statement

The studies involving human participants were reviewed and approved by Ethics Committee, University Hospital Brno. Written informed consent to participate in this study was provided by the participants' legal guardian/next of kin.

## Author Contributions

EH contributed to the trial design, contributed to the study design, participated in clinical data acquisition and analysis, contributed to Supplementary Material preparation, and drafted the manuscript. KP supervised IMP manufacture, contributed to laboratory data acquisition and analysis, contributed to data interpretation, and drafted the manuscript. DC participated in clinical data acquisition, contributed to Supplementary Material preparation, and revised the manuscript. IS performed statistical analysis, contributed to figure preparation and data interpretation, and drafted the manuscript. PMu contributed to the trial design, performed patient enrollment and treatment, contributed to data interpretation, and drafted the manuscript. PMa contributed to the trial design, participated in patient treatment, and drafted the manuscript. LFe contributed to laboratory data acquisition and analysis, contributed to Supplementary Material preparation, and drafted the manuscript. JM contributed to the trial design and drafted the manuscript. LJ participated in IMP manufacturing and revised the manuscript. LSe contributed to IMP manufacturing—monocyte harvest and drafted the manuscript. RP contributed to IMP manufacturing—starting material harvest and revised the manuscript. LFl contributed to IMP manufacturing—certification and revised the manuscript. LSo contributed to the trial design and revised the manuscript. RD, JS, and DV contributed to the trial design, contributed to data interpretation, and revised the manuscript. LZ-D conceived the study design, designed and supervised laboratory data acquisition and analysis, contributed to data analysis and interpretation, and drafted and finalized the manuscript.

### Conflict of Interest

The authors declare that the research was conducted in the absence of any commercial or financial relationships that could be construed as a potential conflict of interest.

## Supplementary Material

Supplementary Figure 1 Decision tree for DC-IMP manufacturing workflow including in-process and quality controls.

Supplementary Figure 2 Number of anti-cancer therapy lines preceding monocyte harvest were compared based on QC parameters: **(A)** DC yield, and post-thaw: **(B)** viability, **(C)** DC phenotype on day 0: CD14, CD197, CD80, CD86, CD83 and on day 2: MHC II, CD80, CD86, CD83 and immunostimulatory properties presented by **(D)** IL-12 production, IL-10 production and IL-12/IL-10 production ratio, **(E)** allo-MLR and auto-MLR.

Supplementary Figure 3 Manufacturing subgroup from monocytes harvested after MTD-based therapy potentially interfering with monocyte biology and manufacturing subgroup from monocytes from untreated patients or after non-interfering treatment compared based on QC parameters: **(A)** DC yield, and post-thaw: **(B)** viability, **(C)** DC phenotype on day 0: CD14, CD197, CD80, CD86, CD83 and on day 2: MHC II, CD80, CD86, CD83 and immunostimulatory properties presented by **(D)** IL-12 production, IL-10 production and IL-12/IL-10 production ratio, **(E)** allo-MLR and auto-MLR.

Supplementary Table 1 Monocyte biology-interfering medications.

Supplementary Table 2 Study patient characteristics, disease course, and therapy.

Supplementary Table 3 Source data: CBC parameters, manufacturing details, and QC parameters.

Supplementary Material 1 html. Interactive—medications 60 days prior to monocyte harvest.
