# Supplementary material for: Dendritic Cell-Based Immunotherapy in Advanced Sarcoma and Neuroblastoma Pediatric Patients: Anti-cancer Treatment Preceding Monocyte Harvest Impairs the Immunostimulatory and Antigen-Presenting Behavior of DCs and Manufacturing Process Outcome
Source: Front Oncol. 2019 Oct 25;9:1034. doi: 10.3389/fonc.2019.01034 (PMC6823179; doi:10.3389/fonc.2019.01034)
Supplement: Supplementary Figure 1 — Decision tree for DC-IMP manufacturing workflow including in-process and quality controls. [file Image_1.pdf]

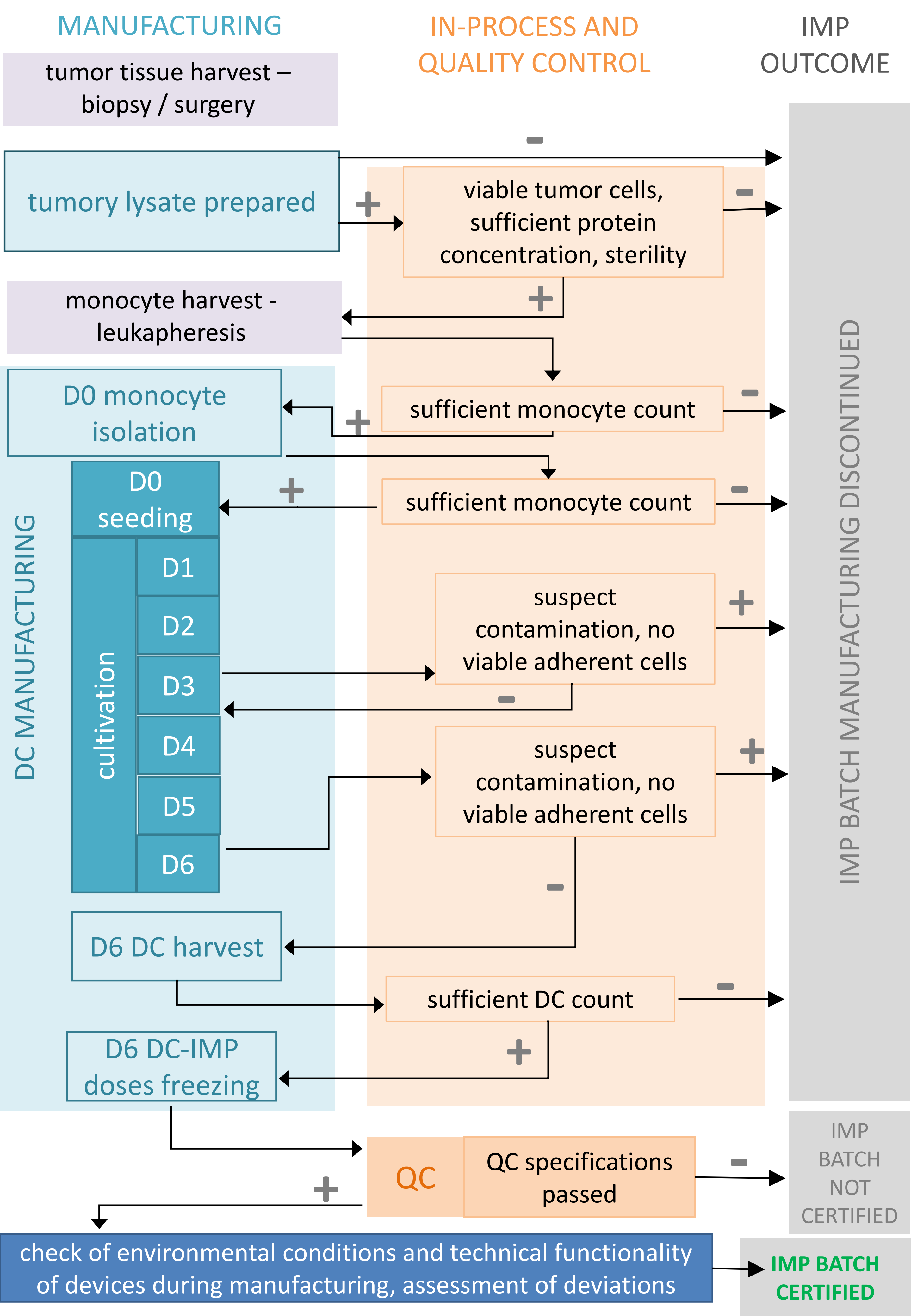
MANUFACTURING
IN-PROCESS AND QUALITY CONTROL
IMP OUTCOME
tumor tissue harvest – biopsy / surgery
tumory lysate prepared
viable tumor cells, sufficient protein concentration, sterility
monocyte harvest - leukapheresis
D0 monocyte isolation
sufficient monocyte count
D0 seeding
sufficient monocyte count
DC MANUFACTURING
cultivation
D1
D2
D3
D4
D5
D6
suspect contamination, no viable adherent cells
suspect contamination, no viable adherent cells
D6 DC harvest
sufficient DC count
D6 DC-IMP doses freezing
QC
QC specifications passed
IMP BATCH MANUFACTURING DISCONTINUED
IMP BATCH NOT CERTIFIED
check of environmental conditions and technical functionality of devices during manufacturing, assessment of deviations
IMP BATCH CERTIFIED

SM2. Figure 1.

Decision tree for DC-IMP manufacturing workflow including in-process and quality controls. Manufacturing of DC-IMP includes tumor lysate and DC manufacturing (blue rectangle). During manufacturing, in-process controls (orange rectangle) are carried out. On the basis of in-process control results, manufacturing is being continued or discontinued (grey rectangle). When manufacturing is completed and sufficient count of DCs is harvested, final product quality control is performed (dark orange rectangle). In the case, the manufactured product does not pass the QC specifications, IMP batch is not certified. If IMP pass the QC specifications, IMP batch is certified after final check of documentation, check of environmental conditions (control of microbial and particle monitoring) and technical functionality of devices (air conditioning, instruments) during manufactuirng and assesment of deviations (dark blue rectangle).
