# Supplementary figures and images for: Dendritic Cell-Based Immunotherapy in Advanced Sarcoma and Neuroblastoma Pediatric Patients: Anti-cancer Treatment Preceding Monocyte Harvest Impairs the Immunostimulatory and Antigen-Presenting Behavior of DCs and Manufacturing Process Outcome
Source: Front Oncol. 2019 Oct 25;9:1034. doi: 10.3389/fonc.2019.01034 (PMC6823179; doi:10.3389/fonc.2019.01034)

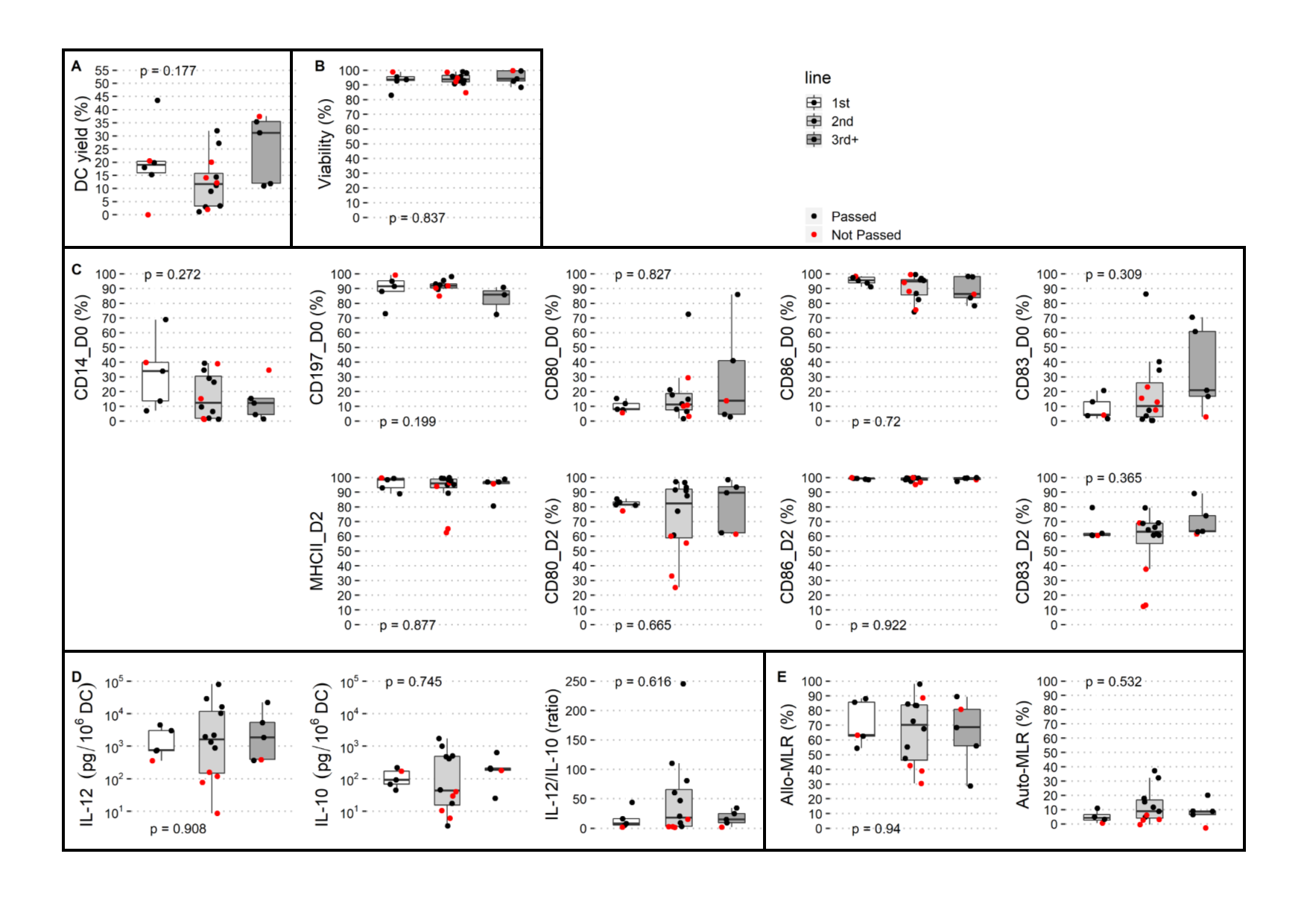

Supplement: Supplementary Figure 2 — Number of anti-cancer therapy lines preceding monocyte harvest were compared based on QC parameters: (A) DC yield, and post-thaw: (B) viability, (C) DC phenotype on day 0: CD14, CD197, CD80, CD86, CD83 and on day 2: MHC II, CD80, CD86, CD83 and immunostimulatory properties presented by (D) IL-12 production, IL-10 production and IL-12/IL-10 production ratio, (E) allo-MLR and auto-MLR. [file Image_2.tif]

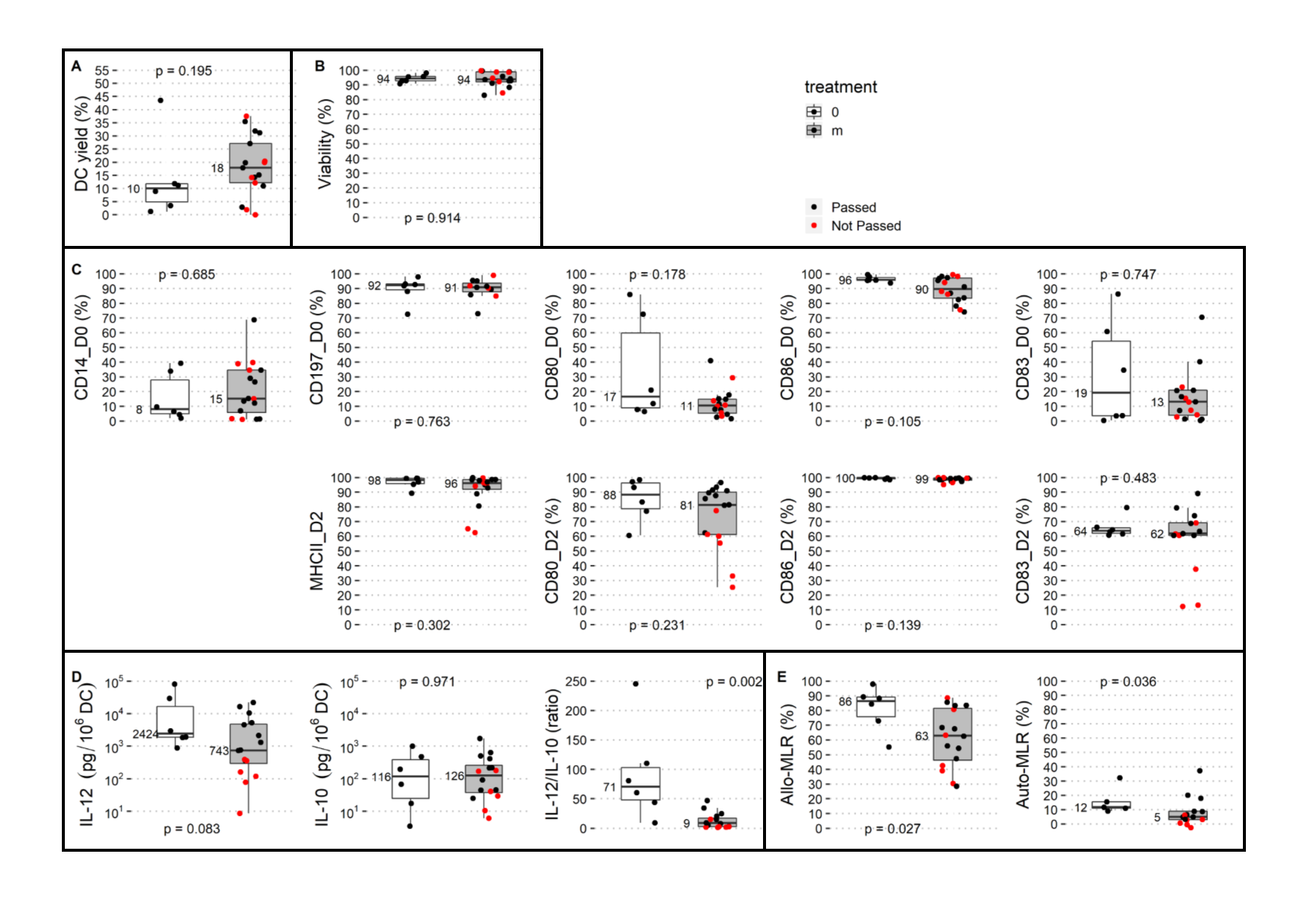

Supplement: Supplementary Figure 3 — Manufacturing subgroup from monocytes harvested after MTD-based therapy potentially interfering with monocyte biology and manufacturing subgroup from monocytes from untreated patients or after non-interfering treatment compared based on QC parameters: (A) DC yield, and post-thaw: (B) viability, (C) DC phenotype on day 0: CD14, CD197, CD80, CD86, CD83 and on day 2: MHC II, CD80, CD86, CD83 and immunostimulatory properties presented by (D) IL-12 production, IL-10 production and IL-12/IL-10 production ratio, (E) allo-MLR and auto-MLR. [file Image_3.tif]
