# Supplementary material for: Dendritic Cell-Based Immunotherapy in Advanced Sarcoma and Neuroblastoma Pediatric Patients: Anti-cancer Treatment Preceding Monocyte Harvest Impairs the Immunostimulatory and Antigen-Presenting Behavior of DCs and Manufacturing Process Outcome
Source: Front Oncol. 2019 Oct 25;9:1034. doi: 10.3389/fonc.2019.01034 (PMC6823179; doi:10.3389/fonc.2019.01034)
Supplement: Supplementary Table 1 — Monocyte biology-interfering medications. [file Table_1.DOCX]

| **Agent** | **Pharmacology Group** | **Potential impact on the generation of DCs from monocytes and on their immunostimulatory properties** (for a particular agent or for a pharmacology group member) | |
| --- | --- | --- | --- |
| Actinomycin D | antineoplastic antibiotic | *Data lacking* | Anticipated effect of transcription inhibitors on DC differentiation |
| Cyclophosphamide | alkylating agents  alkylating-like agents | monocytes and dendritic cells showed *in vitro* higher sensitivity to treatment with the cyclophosphamide analogue mafosfamide than lymphocytes ([Briegert and Kaina, 2007](#_ENREF_2)) | |
| Ifosfamide |  | Impaired the allostimulatory capacity of human dendritic cells by intracellular glutathione depletion ([Kuppner et al., 2003](#_ENREF_11)) | |
| Temozolomide |  | Increased apoptosis and overall cell kill due to monocytes lack of XRCC1 and ligase III alpha expression in the base excision repair pathway ([Briegert and Kaina, 2007](#_ENREF_2)); induced the DNA damage response pathways ATM-Chk2 and ATR-Chk1 in monocytes resulting in p53 activation ([Bauer et al., 2012](#_ENREF_1)) | |
| Cisplatin |  | Monocytes and dendritic cells showed in vitro increased sensitivity to treatment with cisplatin than lymphocytes ([Briegert and Kaina, 2007](#_ENREF_2)) | |
| Denosumab | anti-RANKL | The tumor necrosis factor (TNF)-related cytokine receptor activator of nuclear factor kappa B ligand (RANKL) induced differentiation of hematopoietic precursors common to the monocyte/macrophage and osteoclast lineages into multinucleated, bone-resorbing cells ([Nakagawa et al., 1998](#_ENREF_14)); monocytes functions were not altered by blockade of RANKL function in vivo by RANK-Fc; did not amplify inflammatory processes in mice models of lipopolysaccharide (LPS)–endotoxic shock and inflammatory arthritis ([Seshasayee et al., 2004](#_ENREF_19)) | |
| Filgrastim | recombinant human G-CSF | Reduced whole blood release of TNF‐α, IL‐12, and IFN‐γ in response to endotoxin (lipopolysaccharide) ([Hartung et al., 1999](#_ENREF_5)); showed reduction of phytohemagglutinin‐ or anti‐CD3 antibody–induced lymphocyte proliferation *ex vivo* ([Hartung et al., 1999](#_ENREF_5)) | |
| Methotrexate | folic acid analogue | Displayed dose-dependent antiproliferative and anti-inflammatory effects on human cultured monocytes ([Cutolo et al., 2000](#_ENREF_3)); a strong differentiation factor for immature and undifferentiated monocytic cells ([Seitz et al., 1998](#_ENREF_18)) | |
| Vincristine | vinca alkaloid  antitubulin agent | Altered production of IL-12 by matured DC (mDC), enhanced their production of IL-10, did not affect mDC phenotype (CD83, CD40, CD86, CD58, CD54); promoted apoptotic cell death of mDC ([Rubio et al., 2004](#_ENREF_17)) | |
| Isotretinoid | all trans retinoid acid (ATRA) | Increased apoptotic cell populations in immature DC (imDCs) or mature (mDCs), in the dose-dependent manner suppressed the productions of IL-12p40 and IL-12p70 in imDCs or mDCs, whereas IL-10 was increased ([Jin et al., 2010](#_ENREF_9)) | |
| Temsirolimus | mTOR inhibitor | Temsirolimus – data lacking; sirolimus suppressed chemokines, including MCP-1, RANTES, IL-8, and MIP- 1β in THP-1 cells, and MCP-1, RANTES, IL-8, MIP-1α, and MIP-1β in human primary monocytes via MAPK-p38 and NF-κB-p65 signalling pathways ([Lin et al., 2014](#_ENREF_12)); conditioning human moDCs to skew immune responses towards Th2 can be achieved via an mTOR-dependent and -independent pathway ([Hussaarts et al., 2013](#_ENREF_8)); Rapamycin conditioning of dendritic cells differentiated from human ES cells ([Silk et al., 2012](#_ENREF_20)) or monocytes ([Naranjo-Gomez et al., 2011](#_ENREF_16)) promotes a tolerogenic phenotype; Rapamycin impairs antigen uptake of human dendritic cells ([Monti et al., 2003](#_ENREF_13)) | |
| Sunitinib | tyrosine kinase inhibitors (TKI) | Decreased monocyte count ([Kao et al., 2016](#_ENREF_10)); sunitinib inhibits the expressions of co-stimulatory molecule ligands on dendritic cells ([Ding et al., 2016](#_ENREF_4)) | The VEGF-Receptor Inhibitor Axitinib Impairs Dendritic Cell Phenotype and Function ([Heine et al., 2015](#_ENREF_6)); three MAPK signaling pathways differentially regulate all aspects of phenotypic maturation, cytokine production, and functional maturation of MoDCs ([Nakahara et al., 2006](#_ENREF_15)) |
| Pazopanib |  | In vitro impaired immunostimulatory properties of monocytes including upregulation of immunoinhibitory surface molecule ILT-3 and decreased capability to upregulated MHC II in response to LPS ([Zdrazilova Dubska et al., 2016](#_ENREF_24)) |  |
| Irinotecan | topoisomerase I inhibitor | Moderately inhibited expression of CD14, CD1a and MHC-II on mo DC:, did not induce increased CD86 expression on mo DC cell of compared with control values, inhibited the co-stimulatory molecule CD80 cells expression on moDC ([Hu et al., 2013](#_ENREF_7)) | Camptothecin-induced apoptosis of monocyte-derived dendritic cells ([Valdes-Reyes et al., 2009](#_ENREF_22)) |
| Topotecan |  | Prevented full maturation of DCs stimulated with a cocktail of proinflammatory mediators revealing lower upregulation of NF-κB factors p65 and RelB ([Trojandt et al., 2013](#_ENREF_21)) |  |
| Etoposide | topoisomerase II inhibitor | Decreased monocytes number in peripheral blood in mice ([van't Wout et al., 1989](#_ENREF_23)) | |
| Doxorubicin |  | Inhibited mildly differentiation of moDCs, enhanced the fraction moDC expressing CD80; increased expression of MHC-II on monocytes but did influence CD86 expression ([Hu et al., 2013](#_ENREF_7)) | |

Bauer, M., Goldstein, M., Heylmann, D., and Kaina, B. (2012). Human monocytes undergo excessive apoptosis following temozolomide activating the ATM/ATR pathway while dendritic cells and macrophages are resistant. *PLoS One* 7**,** e39956.

Briegert, M., and Kaina, B. (2007). Human monocytes, but not dendritic cells derived from them, are defective in base excision repair and hypersensitive to methylating agents. *Cancer Res* 67**,** 26-31.

Cutolo, M., Bisso, A., Sulli, A., Felli, L., Briata, M., Pizzorni, C., and Villaggio, B. (2000). Antiproliferative and antiinflammatory effects of methotrexate on cultured differentiating myeloid monocytic cells (THP-1) but not on synovial macrophages from patients with rheumatoid arthritis. *J Rheumatol* 27**,** 2551-2557.

Ding, C., Mai, H., Chen, L., and Zhang, B. (2016). [Sunitinib inhibits the expressions of co-stimulatory molecule ligands on dendritic cells]. *Xi Bao Yu Fen Zi Mian Yi Xue Za Zhi* 32**,** 437-441.

Hartung, T., Doecke, W.D., Bundschuh, D., Foote, M.A., Gantner, F., Hermann, C., Lenz, A., Milwee, S., Rich, B., Simon, B., Volk, H.D., Von Aulock, S., and Wendel, A. (1999). Effect of filgrastim treatment on inflammatory cytokines and lymphocyte functions. *Clin Pharmacol Ther* 66**,** 415-424.

Heine, A., Held, S.A., Daecke, S.N., Riethausen, K., Kotthoff, P., Flores, C., Kurts, C., and Brossart, P. (2015). The VEGF-Receptor Inhibitor Axitinib Impairs Dendritic Cell Phenotype and Function. *PLoS One* 10**,** e0128897.

Hu, J., Kinn, J., Zirakzadeh, A.A., Sherif, A., Norstedt, G., Wikstrom, A.C., and Winqvist, O. (2013). The effects of chemotherapeutic drugs on human monocyte-derived dendritic cell differentiation and antigen presentation. *Clinical and Experimental Immunology* 172**,** 490-499.

Hussaarts, L., Smits, H.H., Schramm, G., Van Der Ham, A.J., Van Der Zon, G.C., Haas, H., Guigas, B., and Yazdanbakhsh, M. (2013). Rapamycin and omega-1: mTOR-dependent and -independent Th2 skewing by human dendritic cells. *Immunology and Cell Biology* 91**,** 486-489.

Jin, C.J., Hong, C.Y., Takei, M., Chung, S.Y., Park, J.S., Pham, T.N., Choi, S.J., Nam, J.H., Chung, I.J., Kim, H.J., and Lee, J.J. (2010). All-trans retinoic acid inhibits the differentiation, maturation, and function of human monocyte-derived dendritic cells. *Leuk Res* 34**,** 513-520.

Kao, J., Timmins, J., Ozao-Choy, J., and Packer, S. (2016). Effects of combined sunitinib and extracranial stereotactic radiotherapy on bone marrow hematopoiesis. *Oncol Lett* 12**,** 2139-2144.

Kuppner, M.C., Scharner, A., Milani, V., Von Hesler, C., Tschop, K.E., Heinz, O., and Issels, R.D. (2003). Ifosfamide impairs the allostimulatory capacity of human dendritic cells by intracellular glutathione depletion. *Blood* 102**,** 3668-3674.

Lin, H.Y., Chang, K.T., Hung, C.C., Kuo, C.H., Hwang, S.J., Chen, H.C., Hung, C.H., and Lin, S.F. (2014). Effects of the mTOR inhibitor rapamycin on monocyte-secreted chemokines. *BMC Immunol* 15**,** 37.

Monti, P., Mercalli, A., Leone, B.E., Valerio, D.C., Allavena, P., and Piemonti, L. (2003). Rapamycin impairs antigen uptake of human dendritic cells. *Transplantation* 75**,** 137-145.

Nakagawa, N., Kinosaki, M., Yamaguchi, K., Shima, N., Yasuda, H., Yano, K., Morinaga, T., and Higashio, K. (1998). RANK is the essential signaling receptor for osteoclast differentiation factor in osteoclastogenesis. *Biochemical and Biophysical Research Communications* 253**,** 395-400.

Nakahara, T., Moroi, Y., Uchi, H., and Furue, M. (2006). Differential role of MAPK signaling in human dendritic cell maturation and Th1/Th2 engagement. *J Dermatol Sci* 42**,** 1-11.

Naranjo-Gomez, M., Raich-Regue, D., Onate, C., Grau-Lopez, L., Ramo-Tello, C., Pujol-Borrell, R., Martinez-Caceres, E., and Borras, F.E. (2011). Comparative study of clinical grade human tolerogenic dendritic cells. *J Transl Med* 9**,** 89.

Rubio, M.T., Ittelet, D., Raymond, E., Blay, J.Y., Bernard, J., and Chouaib, S. (2004). The immunosuppressive effect of vincristine on allostimulatory potential of human dendritic cells interferes with their function and survival. *Int J Oncol* 25**,** 407-412.

Seitz, M., Zwicker, M., and Loetscher, P. (1998). Effects of methotrexate on differentiation of monocytes and production of cytokine inhibitors by monocytes. *Arthritis Rheum* 41**,** 2032-2038.

Seshasayee, D., Wang, H., Lee, W.P., Gribling, P., Ross, J., Van Bruggen, N., Carano, R., and Grewal, I.S. (2004). A novel in vivo role for osteoprotegerin ligand in activation of monocyte effector function and inflammatory response. *Journal of Biological Chemistry* 279**,** 30202-30209.

Silk, K.M., Leishman, A.J., Nishimoto, K.P., Reddy, A., and Fairchild, P.J. (2012). Rapamycin conditioning of dendritic cells differentiated from human ES cells promotes a tolerogenic phenotype. *J Biomed Biotechnol* 2012**,** 172420.

Trojandt, S., Knies, D., Pektor, S., Ritz, S., Mailander, V., Grabbe, S., Reske-Kunz, A.B., and Bros, M. (2013). The chemotherapeutic agent topotecan differentially modulates the phenotype and function of dendritic cells. *Cancer Immunol Immunother* 62**,** 1315-1326.

Valdes-Reyes, L., Argueta, J., Moran, J., Salaiza, N., Hernandez, J., Berzunza, M., Aguirre-Garcia, M., Becker, I., and Gutierrez-Kobeh, L. (2009). Leishmania mexicana: inhibition of camptothecin-induced apoptosis of monocyte-derived dendritic cells. *Experimental Parasitology* 121**,** 199-207.

Van't Wout, J.W., Linde, I., Leijh, P.C., and Van Furth, R. (1989). Effect of irradiation, cyclophosphamide, and etoposide (VP-16) on number of peripheral blood and peritoneal leukocytes in mice under normal conditions and during acute inflammatory reaction. *Inflammation* 13**,** 1-14.

Zdrazilova Dubska, L., Fedorova, L., Pilatova, K., Mudry, P., Hlavackova, E., Matoulkova, E., Flajsarova, L., Demlova, R., Valik, D., and Sterba, J. (2016). TKI pazopanib impaires immunostimulatory properties of monocytes: Implication for monocyte-derived DC-based anti-cancer vaccine preparation. *ANNALS OF ONCOLOGY* 27.
