# Supplementary material for: Dendritic Cell-Based Immunotherapy in Advanced Sarcoma and Neuroblastoma Pediatric Patients: Anti-cancer Treatment Preceding Monocyte Harvest Impairs the Immunostimulatory and Antigen-Presenting Behavior of DCs and Manufacturing Process Outcome
Source: Front Oncol. 2019 Oct 25;9:1034. doi: 10.3389/fonc.2019.01034 (PMC6823179; doi:10.3389/fonc.2019.01034)
Supplement: Supplementary Table 2 — Study patient characteristics, disease course, and therapy. [file Table_2.DOCX]

| **Subject No./ Date of primary dg /Age at dg/ Primary dg** | **Disease course** | **Anti-cancer therapy**  **(CTx, RTx, ITx)** | **Date of mono-**  **nuclear harvest/**  **Age at harvest/ PS at harvest** | **Thx 60 days prior to mononuclear harvest** | **Date of DC ITx initiation/ Stage of the disease at DC ITx initiation** | |
| --- | --- | --- | --- | --- | --- | --- |
| KDO-0101  11/2011  10 years  Ewing sarcoma of the mandible  EWS/FLI1 posit  Mts parietal bone | 1^st^ relapse 09/2015, mts parietal bone  2^nd^ relapse 06/2017 locoregional relapse with mts progression - pelvis, spine, cranial bones, liver  3^rd^ progression 06/2018  mts - pelvis, spine, cranial bones, liver | 1^st^ line CTx - Euro Ewing 2008, 12/2011 - 10/2012  Partial resection - primary tumor, 06/2012, 1^st^ PR  HD CTx Treo/Mel + APBSCT, 07/2012  RTx mandible + parietal bone, 34 Gy + 45 Gy,  09/2012 - 11/2012  Euro Ewing 2008, maintenance CTx, 7 x VAC,  10/2012- 05/2013, 1^st^ CR  Resection - mts parietal bone, 09/2015 , 2^nd^ CR  2^nd^ line CTx - VCR/Irino + Pazopanib, 09/2015 - 04/2016  RTx cranial bones 41 Gy, 01/2016 - 02/2016  HD CTx Treo/Mel + APBSCT, 04/2016  CPM, 09/2016 - 06/2017  Experimental ITx - 1^st^ DC vac course, 19 doses  08/2016 - 06/2017  Resection – mandible, 06/2017  3^rd^ line CTx - Topo/CPM + Zoledronic acid,  07/2017 - 09/2017, SD  RTx of the mandible, cranial bones, pelvis - 21 Gy  08/2017 - 09/2017  4^th^ line CTx VBL weekly+Metformin, 01/2018 - 06/2018, SD  Nivolumab every 2 weeks, 02/2018 - 06/2018  Experimental ITx: 2^nd^ DC vac course, 03/2018 - 11/2018, PD | 07.01.2016  15 years  KS 100 | D60 -> D0  Pazopanib 200 mg/day; TMP/SMT 920 mg/day/twice weekly  D58 -> D54  CTx cycle: VCR/Irino  D58 VCR 2 mg/day  D58 -> D54 Irino 76 mg/day  D51 VCR 2 mg/day  D44 VCR 2 mg/day  D44 -> D37 Filgrastim 240 µg/day  D30 -> D24 Cefixime 400 mg/day | 29.08.2016  2^nd^ CR  KS 100 | |
| KDO-0102  05/2012  6 years  Localized  high-grade osteosarcoma of the right distal femur | 1^st^ relapse 04/2014  mts of lungs and left tibia  2^nd^ progression  11/2015  mts lungs bilat  3^rd^ progression  06/2016  mts of lungs bilat, thorax, abdomen | 1^st^ line CTx - AOST 0331, 05/2012 - 02/2013  Resection - primary tumor, 08/2012, 1^st^ CR  Mifamurtid 10/2012 - 04/2013  Tibial biopsy 04/2014  2^nd^ line CTx - Ifo/Eto + zoledronic acid  06/2014 - 01/2015, PR  3^rd^ line CTx - COMBAT III + Pazopanib,  01/2015 - 09/2015, PD  Biopsy - lung mts, 10/2015  4^th^ line CTx - rechallenge therapy  2 cycles HD MTX 10/2015 - 11/2015, PD  5^th^ line CTx - VBL /CPM + Sunitinib,  12/2015 - 02/2016  RTx lungs + left calf, 15 Gy + 36 Gy, 01/2016 - 02/2016  Nivolumab - 4 doses, 03/2016 - 04/2016, PD  RTx - lungs, 17.5 Gy, 06/2016  Experimental ITx - DC vac course, 17 doses  01/2016 - 09/2016 | 01.12.2015  10 years  LS 90 | D60 -> D0 TMP/SMT  920 mg/day/twice weekly  D46 MTX 12g/day  D45 -> D43 Ca-folinate 60 mg/day  D34 MTX 12 g/day  D33 -> D31 Ca-folinate 60 mg/day | 12.01.2016  PD | |
| KDO-0103  06/2015  13 years  Alveolar rhabdomyosarcoma of the right calf  Mts regional and supraregional lymph nodes | CR not achieved  continuous progression | 1^st^ line CTx - ARST08P1 + Pazopanib,  07/2015 - 10/2015  Resection - primary tumor, 10/2015  2^nd^ line CTx - ARST 0921 + TEM,  11/2015 - 01/2016  RTx - primary tumor + regional lymph nodes, 34.4 Gy, 11/2015, PD  COMBAT III modifed + nivolumab,  01/2016 - 07/2016  Experimental ITx - DC vac course, 4 doses  02/2016 - 03/2016 | 14.12.2015  14 years  KS 90 | D60 -> D0 TMP/SMT 1320 mg/day/twice weekly  Mirtazapinum 30 mg/day  LMWH 3800 IU/day  Pregabalin 300 mg/day  D50 -> D40 Clindamycin 1200 mg/day  D38 -> D37 CTx cycle VDC + TEM  D38 TEM 23.4 mg/day  D38 VCR 2 mg/day  D38 -> D37 Doxo 44 mg/day  D38 CPM 1872mg/day  D33 -> D24 Filgrastim 250 µg/day  D31 VCR 2 mg/day,  D31 TEM 23.4 mg/day  D25 -> D20 RTx, total dose: 34.4 Gy  D21 -> D17 Ifo 2808 mg/day  D21 -> D17 Eto 156 mg/day  D16 -> D7 Filgrastim 250 uq/day  D16 Triptorelin 11.25 mg | 09.02.2016  PD  KS 80 | |
| KDO-0109  01/2014  10 years  Localized Ewing sarcoma of the left femur  EWS/FLI1+ | 1^st^ relapse 02/2016  mts bones, lungs, bone marrow  . | 1^st^ line CTx - EuroEwing 2008, 02/2014-12/2014  reductions of IFO and ETO due to toxicities  Resection - primary tumor, 09/2014  RTx - primary tumor, 29 Gy, 12/2014-01/2015  1^st^ CR  2^nd^ line CTx - AEWS1031, 02/2016 - 03/2016, PD  3^rd^ line CTx - ARST08P1 + Sunitinib,  03/2016 - 06/2016  Palliative RTx of Th6, 28Gy, 04/2016, PR | 24.03.2016  13 years  KS 90 | D60 -> D0 TMP/SMT 720 mg/day/twice weekly  Pazopanib 200 mg/2 day  LMWH 5700 IU/day  D47 -> D35 AMX/CLA 1875 mg/day  D35 -> D31 CTx cycle VTC  D35 VCR 2 mg/day  D35 -> D31 Topo 1.22 mg/day  D35 -> D31 CPM 407.5 mg/day  D29 VCR 2 mg/day  D10 -> D5 Acyclovir 2000 mg/day | Not treated | |
| KDO-0111  11/2013  8 years  Localized Ewing sarcoma of the left distal humerus | 1^st^ locoregional relapse  02/2016 | 1^st^ line CTx - EuroEwing 2008, 12/2013 - 10/2014  (Ifo neurotoxicity Gr.IV - switch to CPM)  Resection - primary tumor, 05/2014  1^st^ CR  Biopsy - locoregional relapse, 02/2016  2^nd^ line CTx - AEWS1031 + Votrient,  02/2016-08/2016  RTx - primary tumor, 54Gy, 08/2016-09/2016  Resection - locoregional relapse, 09/2016 , 2^nd^ CR  HD CTx Treo/Mel 10/2016  Individualized CTx - Sunitinib every second day  12/2016-04/2018  CYC week on/week off 04/2017-2/2019 | 09.05.2016  12 years  KS 100 | D60 -> D0 TMP/SMT 1920 mg/day/twice weekly  D60 -> D7 Pazopanib 200 mg/day  D45 -> D41 CTx cycle VTC  D45 VCR 2 mg/day  D45 -> D41 Topo 1.25 mg/day  D45 -> D41 CPM 417.5 mg/day  D39 VCR 2 mg/day  D36 -> D30 Filgarstim 300 µg/day  D19 -> D15 CTx cycle VTC  D19 VCR 2 mg/day  D19 -> D15 Topo 1.26 mg/day  D19 -> D15 CPM 422.5 mg/day  D13 -> D7 Filgarstim 300 µg/day  D12 VCR 2 mg/day | Not treated | |
| KDO-0114  12/2014  13 years  Synovial sarcoma of the left thigh  Mts lungs and retroperitoneum | 1^st^ progression  10/2016  mts left hemithorax | 1^st^ line CTx - ARST1321 + Metformin + Pazopanib  12/2014 - 05/2015  RTx - primary tumor 45Gy, 03/2015  Resection - primary tumor, 05/2015  COMBAT III 05/2015 - 12/2016  Thoracoscopic exstirpation of mts of the left hemithorax 10/2016  2^nd^ line CTx - ARST08P1 – 1 cycle IE, 12/2016  Palliative RTx - left hemithorax 15 Gy, 01/2017  Palliative experimental thx valproate, metformin,  Lipanthyl, sunitinib 12/2016 - 06/2017  Experimental ITx - DC vac course, 11 doses  01/2017 - 06/2017 | 12.12.2016  15 years  KS 80 | D60 -> D0  Carvediol 37 mg/day  Fenofibrate 160 mg/day  Metformin 500 mg/day  Cholecalciferol  2000 IU/day  D60 -> D55, D50 -> D7  CPM 50 mg/day  D4 -> D0 AMX/CLA 2 g/day | 18.01.2017  PD  KS 80 | |
| KDO-0115  03/2015  10 years  Neuroblastoma of the retroperitoneum  Skeletal mts, bone marrow infiltration | 1^st^ progression 04/2016  2^nd^ progression 11/2016 | 1^st^ line CTx - ANBL 0532, 03/2015-10/2015  poor response after 4cycles MATIN  1^st^ application 10.08.2015  2^nd^ application 24.08.2015  HD CTx Bu/Mel + APBSC, 10/2015  RTx - primary tumor 26.4 Gy, 12/2015 - 01/2016  Retinoic acid 02/2016 - 05/2016, 4 cycles, PD  2^nd^ line CTx - METRO-NB2012, 05/2016 -10/2016  6 cycles, PD  3^rd^ line CTx - VBL, Celecoxib, Endoxan,Propranolol  11/2016 - 06/2017  Experimental ITx - DC vac course, 9 doses  11/2016 - 03/2017  Palliative RTx - mts cranial bones 12 Gy, 02/2017 | 30.06.2016  11 years  KS 80 | D60 -> D0 TMP/SMT 720 mg/day/twice weekly  D44 -> D0  Celecoxib 400 mg/day  Propranolol 50 mg/day  D44 -> D28  CPM 25 mg/day  Eto 13.25 mg/day  D30 VBL 1 mg/day | 16.11.2016  PD  KS 80 | |
| KDO-0118  06/2005  13 years  Localized Ewing sarcoma of the spine C5-Th2  EWS/FLI1 negat  Extradural involvement | 1^st^ locoregional relapse  08/2016  EWSR1 gene disruption confirmed  2^nd^ locoregional progression 04/2017 | 1^st^ line CTx - Euro Ewing 99, 07/2005 - 12/2005  CR  HD CTx + APBSC 01/2006  RTx - primary tumor 49.2 Gy, 03/2006 - 04/2006  2^nd^ line CTx - AEWS1031, 08/2016 - 02/2017  2 cycles VTC - SD  2 cycles VCR/IRINO - PR | 05.01.2017  24 years  KS 100 | D60 -> D0 TMP/SMT 1920 mg/day/twice weekly  D60 -> D40  Sunitinib 25 mg/day  D48 -> D44 CTx cycle  VCR/Irino  D48 VCR 2 mg/day  D48 -> D44  Irino 78 mg/day | 20.2.2017  PR  KS 100 | |
| KDO-0119  10/2016  12 years  Alveolar rhabdomyosarcoma  Primum ignotum  Mts bones and bone marrow | Without relapse | 1^st^ line CTx - ARST08P1 + TEM, 10/2016 - 05/2018  TEP left 11/2016, TEP right 12/2016  Maintenance CTx - EpSSG RMS 2005, VNR + CPM  05/2018 – ongoing  Experimental ITx - DC vac course, 22 doses  4/2018 – ongoing | 03.04.2017  12 years  KS 70 | D60 -> D28, D13 -> D1  LMWH 2000 IU/day  D60 -> D0 TMP/SMT 720 mg/day/twice weekly  D51 -> D50 CTx cycle VDC  D51 VCR 1.74 mg/day  D51 -> D50 Doxo 43.5 mg/day  D51 CPM 1392 mg/day  D51 TEM 11.6 mg  D50 Zoledronic acid 4 mg/day  D32 VCR 1.74 mg/day  D32 TEM 11.6 mg/day  D28 -> D18 Pip/tazo 9.6 g/day,  D28 -> D18 Ami 420 mg/day  D25 -> D18 VAN 1.2 g/day  D25 -> D18 AmphoB 160 mg/day  D18 -> D13 AMX/CLA - 1 g/day  D18 -> D13 Cipro 500 mg/day  D9 -> D8 Filgrastim 150 µg/day | 11.04.2018  PR  KS 80 | |
| KDO-0120  09/2016  8 years  High-grade osteoblastic osteosarcoma of the left distal femur  Mts lungs bilat | Without relapse | 1^st^ line CTx - AOST 0331, 10/2016 - 07/2017  Resection - primary tumor, 03/2017  1^st^ CR  COMBAT III 11/2017 – ongoing | 16.03.2017  8 years  KS 80 | D60 -> D0 TMP/SMT  720 mg/day/twice weekly  Citalopram 10 mg/day  Itopridi hydrochloridum 150 mg/day  D54 MTX 11 g/day  D53 -> D-50 Ca-folinate 60 mg/day  D44 MTX 11 g/day  D43 -> D40 Ca-folinate 60 mg/day  D34 -> D33 CTx cycle  D34 -> D33 Doxo 34 mg/day  D34 -> D33 Cisplatin 54 mg/day | Not treated | |
| KDO-0121  11/2016  14 years  Ewing sarcoma of the pelvis,  EWS/FLI1 +  Mts - bones, bone marrow, lungs, meninges | 1^st^ progression  06/2017  mts - bones, bone marrow | 1^st^ line CTx - Euro Ewing 2008, 11/2016 - 05/2017  - after 5th VIDE VGPR, only residual PET positivity in the primary tumor  Biopsy - residual tumor, 05/2017, 20% viability | 01.06.2017  15 years  KS 80 | D60 -> D0 Sertraline 50 mg/day  D60 -> D0 TMP/SMT 720 mg/day/twice weekly  D48 -> D46 CTx cycle VIDE  D48 VCR 0.22 mg/day  D48 -> D46 Ifo 3840 mg/day  D48 -> D-46 Doxo 25.6 mg/day  D48 -> D46 Eto 192 mg/day  D35 -> D30 Filgrastim 175 µg/day  D34 Zoledronic acid 4 mg/day  D18 Triptorelin 11.25 mg | Not treated | |
| KDO-0122  08/2014  13 years  Ewing sarcoma of the left proximal tibia, EWS/FLI1+  Mts of lungs | 1^st^ relapse 10/2016  skip mts of left proximal femur,  mts of lungs  2^nd^ relapse 07/2018  mts - left thoracal wall, pelvis  3^rd^ progression  12/2018  mts -vertebrae, pelvis, bone marrow, lungs | 1^st^ line CTx - Euro Ewing 2008, 08/2014 - 07/2015  Resection - primary tumor, 02/2015,  1^st^ CR  RTx - lungs 18 Gy, 07/2015  Biopsy - femur mts 12/2016  2^nd^ line CTx - VTC cycles, 01/2017 - 05/2017  Radical resection - tumor residuum, 05/2017  HD CTx Treo/Mel 06/2017,  2^nd^ CR  Post-consolidation maintenance therapy  VBL, Celecoxib, CPM, 10/2017 - 07/2018  Biopsy - left thoracal wall 07/2018  3^rd^ line CTx - 3x Irino/TZM, 08/2018 - 11/2018  PD  4^th^ line Thx - Experimental ITx: 2^nd^ DC vac course,  2 doses, 12/2018  Experimental Thx olaparib + nivolumab  Palliative RTx 20 Gy, left hemithorax, 01/2019 | 23.03.2017  15 years  KS 100 | D60 -> D0 LMWH 3800 IU/day  TMP/SMT 1920 mg/twice weekly  Fentanyl 25 µg/h  D60 -> D51 Filgrastim 480 µg/day  D44 -> D40 CTx cycle VTC  D44 VCR 2 mg/day  D44 -> D-40 Topo 1.5 mg/day  D44 -> D40 CPM 497.5 mg/day  D39 -> D28 Filgrastim 640 µg/day  D35 -> D27 Pip/Tazo 13.5 g/day  D35 -> D27 Amikacin 1.3 g/day  D21 -> D17 CTx cycle VTC  D21 VCR 2 mg/day  D21 -> D17 Topo 1.5 mg/day  D21 -> D17 CPM 497.5 mg/day  D16 -> D5 Filgrastim 480 µg/day  D8 -> D2 Pip/Tazo 13.5 g/day  D8 -> D2 Amikacin 1.3 g/day | Not treated | |
| KDO-0124  12/2012  14 years  Localized  osteoblastic osteosarcoma of the right proximal tibia | 1^st^ relapse 09/2014  mts of lungs bilat  2^nd^ relapse 01/2017  mts of lungs | 1^st^ line CTx - AOST 0331, 12/2012 - 11/2013  Resection - primary tumor + TEP 03/2013, 1^st^ CR  Resection - lung mts 11/2014, 2^nd^ CR  2^nd^ line CTx - 4 x Ifo/Eto, 02/2015 - 04/2015  COMBAT III 05/2015 - 01/2017  Resection - lung mts 02/2017  3^rd^ line CTx - AOST 1321 + VBL + CPM,  02/2017 - 10/2017  Experimental ITx - DC vac course, 16 doses  05/2017 - 12/2017 | 20.03.2017  19 years  KS 100 | D60 -> D0 Sertraline 50 mg/day  Levocetirizin 5 mg/day  Montelucast 10 mg/day  Budesonide/Formoterol 400 µg/day  D33 -> D0 TMP/SMT 1920 mg/day/twice weekly  D34 -> D26 CPM 50 mg/day  D34, D27, D20 VBL 4.8 mg/day  D34, D27, D20  Denosumab 120 mg/day | 05.05.2017  2^nd^ mts relapse  PR after resection  KS 100 | |
| KDO-0131  08/2017  18years  Embryonal rhabdomyo-sarcoma of the pelvis  Mts of the prostate, seminal tubes, rectum, obturator muscles, penis, skeletal mts | Without relapse | 1^st^ line CTx - EpSSG RMS 2005, 09/2017 - 06/2018  Experimental ITx - DC vac course, 18 doses  06/2018 – ongoing | 11.01.2018  18 years  KS 90 | D60 -> D0  Sertraline 50 mg/day  Gabapentin 600 mg/day  Ranitidin 75 mg/day TMP/SMT 960 mg/day/twice weekly  D37 -> D36 CTx cycle IVA  D37 VCR 1.8 mg/day  D37 Actino 1.8 mg/day  D37 -> D36  Ifo 5400 mg/day | 08.06.2018  PR  KS 70 | |
| KDO-0133  10/2011  16 years  Localized  osteosarcoma of the right proximal femur | 1^st^ relapse 06/2015  lung mts  2^nd^ relaps 06/2016  lung mts  3^rd^ relapse 01/2018  lung mts  4^th^ relapse 12/2018  Lung mts | 1^st^ line CTx - AOST 0331, 10/2011 - 07/2012  Resection - primary tumor 01/2012, 1^st^ CR  RTx primary tumor 54 Gy, 02/2012  Resection - lung mts 06/2015, 2^nd^ CR  Resection - lung mts 06/2016, 3^rd^ CR  2^nd^ line CTx - AOST 1321, 08/2016 - 12/2017  + Sunitinib from 11/2016  + Metformin from 10/2017  RTx lungs 15 Gy, 02/2017 - 03/2017  Resection - lung mts 02/2018, 4^th^ CR  3^rd^ line CTx - COMBAT III, 04/2018 - 12/2018  Experimental ITx - 1^st^ DC vac course, 6 doses  06/2018 - 09/2018  2^nd^ DC vac course, 6 doses, 12/2018 - 2/2019  Resection - lung mts, 12/2018, 5^th^ CR | 09.04.2018  24 years  KS 90 | D60 -> D0  Budesonide/formoterol  1 breath/day  Sertraline 50 mg/day  Digoxin 0.125 mg/day  Enalapril 5 mg/day  Bisoprolol 1.25 mg/day | 13.06.2018  PD  KS 100 |  |
| KDO-0135  11/2017  3 years  High-risk neuro-  blastoma of the left suprarenal gland  Mts bones, bone marrow, liver, cervical lymph nodes | Without relapse | 1^st^ line CTx - ANBL0532, 11/2017 - 03/2018, SD  Partial resection - primary tumor 02/2018  MATIN 04/2018, 1 cycle - not well tolerated, PR  HD CTx + APBSC, 05/2018, PR  RTx retroperitoneum + skeletal mts 45 Gy, 08/2018, PR  Imunotherapy - anti-GD2 + retinoic acid  11/2018 - 02/2019, PR | 11.02.2019  5 years  LS 100 | D60 -> D0 TMP/SMT  240 mg/day/twice weekly  D60 -> D50 Dinutxmb 7.3 mg/day  D50 -> D36, Retinoic acid 120 mg/day  D30 Gabapentin 200 mg/day  D29 Gabapentin 400 mg/day  D28 -> D18 Gabapentin 600 mg/day  D28 -> D23 Dinutxmb 7.3 mg/day  D23 -> D18 Dinutxmb 7.3 mg/day  D18 -> D4 Retinoic acid 120 mg/day | Pending  NA |  |
| KDO-0137  08/2016  16 years  Localized  synovial sarcoma of the neck | 1^st^ relapse  02/2018  Mts spine, pelvis, lungs  2^nd^ progression  07/2018 skeletal mts  3^rd^ progression  09/2018  skeletal mts  4^th^ progression  12/2018 skeletal mts | 1^st^ line CTx- Ifo/Doxo, 08/2016 - 01/2017  RTx - primary tumor 50.4 Gy, 12/2016 - 01/2017  1^st^ CR  2^nd^ line CTx - Modified COMBAT III, 04/2018 - ongoing  + Pazopanib starting 08/2018  Palliative RTx: Th1 5x2.5 Gy, Th4 and Th8 and L2 5x4 Gy, pelvis l.sin 8x3 Gy, 07/2018 - 08/2018  Biopsy - pelvis mts 07/2018  Experimental ITx - DC vac course, 2 doses  07/2018 - 08/2018  Re-biopsy - mts os sacrum 10/2018 | 11.06.2018  18 years  KS 90 | D60 -> D0 TMP/SMT 1920 mg/day/twice weekly  D58 -> D0  Celecoxib 600 mg/day  Fenofibrate 267 mg/day  D58 -> D7 CPM 50 mg/day  D58, D51, D44, D37,  D30, D23, D16, D9  VBL 3.96 mg/day | 30.07.2018  PD  KS 90 |  |
| KDO-0139  09/2016  20 years  High-grade osteoblastic osteosarcoma of the left distal femur  Solitary lung mts | 1^st^ relapse  05/2018  mts of lungs bilat | 1^st^ line CTx - AOST0331, 09/2016 - 05/2017  Resection - primary tumor 12/2016  1^st^ CR  Resection - lung mts 07/2018  2^nd^ line CTx - AOST0331 – cycle IE 07/2018  PR  RTx - lung mts 15Gy, 08/2018  3^rd^ line CTx - COMBAT III modified, 10/2018- 02/2019  + Olaparib 01/2019 - 02/2019  Experimental ITx - DC vac course, 6 doses  11/2018 - 02/2019 | 24.09.2018  22 years  KS 100 | D53 -> D49 CTx cycle Ifo/Eto  D53 -> D49 Ifo 4704 mg/day  D53 -> D49 Eto 168 mg/day  D53 -> D49 Uromitexan 5 g/day  D53 -> D49 Flonidan 10 mg/day  D51 -> D40, D30 -> D28  RTx lungs, total dose 15 Gy  D48 -> D0 TMP/SMT 1920 mg/day/twice weekly  Fludrocortison 0.2 mg/day  D48 -> D38 Filgrastim 300 µg/day  D40 -> D32 Cefepime 8 g/day  D40 -> D32 Amikacin 7.2 g/day  D33 -> D29 CTx cycle Ifo/Eto  D33 -> D29 Eto 165 mg/day  D33 -> D29 Ifo 4620 mg/day  D33 -> D29 Uromitexan 5 g/day  D33 -> D29 Flonidan 10 mg/day  D40 Triptorelin 11.25 mg | 28.11.2018  SD  KS 90 |  |
| KDO-0141  09/2015  18 years  Localized  synovial sarcoma of the left calf | 1^st^ relapse 06/2018  Mts lungs | 1^st^ line CTx - 3 cycles Ifo/Doxo, 09/2015 - 11/2015, SD  Amputation - left femur 12/2015  1^st^ CR  Metronomic therapy: CPM+metformin, 03/2016 - 06/2018  Resection - lung mts 08/2018  2^nd^ line CTx - COMBAT III modified, 08/2018 - 02/2019, + Crizotinib 10/2018 - 02/2019  RTx - lungs 18 Gy, 10/2018 - 11/2018  Experimental ITx - DC vac course, 5 doses  12/2018 - 02/2019 | 18.10.2018  22 years  KS 70 | D60 -> D0 TMP/SMT 1920 mg/day/twice weekly  Betaxolol 20 mg/day  Cortisol 9 mg/day  D50, D42, D35, D28, D21, D14  VBL 4 mg/day  D42 -> D7 Eto 25 mg/day  D50 -> D0 Celecoxib 800 mg/day  Cholecalciferol 250 IU/day  Fenofibrate 267 mg/day | 21.12.2018  SD  KS 70 |  |
| KDO-0142  09/2017  2 years  Neuroblastoma of the right retroperitoneum  Mts bones, bone marrow | 1^st^ locoregional progression  7/2018  2^nd^locoregional progression  10/2018 | 1^st^ line CTx - ANBL0532, 09/2017- 03/2018  Subtotal resection - primary tumor 01/2018  HD CTx Bu/Mel 03/2018  RTx retroperitoneum 30.6 Gy,  04/2018 - 05/2018  Retinoic acid - 1 cycle 06/2018  2^nd^ line CTx - ANBL 1221 - 3 cycles T/Irino + Dinutxmb (antiGD2), 08/2018 - 11/2018  3^rd^ line CTx - Topo/CPM, 11/2018 - 01/2019  Nivolumab 01/2019 - ongoing | 27.08.2018  3 years  KS 100 | D60 -> D0 TMP/SMT 960 mg/day/twice weekly  D27 -> D23 Irino 30.5 mg/day  D27 -> D23 TZM 61 mg/day  D32 Gabapentin 200 mg/day  D31 Gabapentin 400 mg/day  D30 -> D16 Gabapentin 600 mg/day  D26 -> D21 Dinutxmb 6.5 mg/day  D21 -> D16 Dinutxmb 6.5 mg/day | Not treated |  |
| KDO-0143  05/2004  1 year  Localized  embryonal rhabdomyoma-  sarcoma of pelvis | 1^st^ locoregional relapse  02/2014  2^nd^ locoregional relapse  05/2018  3^rd^ locoregional progression  01/2019 | 1^st^ line CTx - 2x VCR/Irino, 6x IVA, 05/2004 - 01/2005  Resection – pelvic tumor residuum 10/2004, 1^st^ CR  Biopsy - pelvic relapse 02/2014  2^nd^ line CTx - 7x VCR/Irino/TZM, 02/2014 - 09/2014  Resection – pelvic tumor residuum, 06/2014, 10/2014  RTx pelvis 50.4 Gy 12/2014 - 01/2015  Maintenance CTx - Vinorelbine + CPM  10/2014 - 10/2015, 2^nd^ CR  Resection - radical cystectomy, hysterectomy, colpectomy, bilateral urethrostomy 07/2018  3^rd^ line CTx - rEECur - Topo/CYC, 08/2018 - 12/2018  Biopsy – pelvic residuum + transversostomy 01/2019  Palliative RTx pelvis 18 Gy, 01/2019  4^th^ line CTx - ARST 0921: Vinorelbine/CPM 02/2019 | 13.09.2018  15 years  KS 100 | D41 -> D0 LMWH 5700 IU/day  D43, D15 Pentamidine 220 mg/day  D41 -> D37 CTx cycle Topo/CPM  D41 -> D37 Topo 1.19 mg/day  D41 -> D37 CPM 397.5 mg/day  D21 -> D17 CTx cycle Topo/CPM  D21 -> D17 Topo 1.19 mg/day  D21 -> D17 CPM 397.5 mg/day  D28 -> D21 Acyclovir 2 g/day  D28 -> D22 Cefepime 4 g/day  D28 -> D22 Amikacin 850 mg/day | Pending  NA |  |
| KDO-0144  10/2014  18 years  Localized  Ewing sarcoma of the left tibia  EWS/FLI1+ | 1^st^ relapse  07/2018  mts lungs | 1^st^ line CTx - EuroEwing 2008, 11/2014 - 11/2015  Resection - primary tumor 05/2015, 1^st^ CR  Resection - lung mts 08/2018  2^nd^ line CTx - 2x Irino/TZM, 08/2018 - 10/2018  SD  3^rd^ line CTx – rEECur: Topo/CPM, 10/2018 - 12/2018  4^th^ line CTx - modified COMBAT III, 01/2019  + Valproate 02/2019 | 22.10.2018  22 years  KS 100 | D54 -> D0 TMP/SMT 1920 mg/day/twice weekly  D54 -> D50 CTx cycle Irino/TZM  D54 -> D50 TZM 200 mg/day  D54 -> D50 Irino 95 mg/day  D36 -> D27 Cefixime 800 mg/day  D34 -> D30 CTx cycle Irino/TZM  D34 -> D30 TZM 200 mg/day  D34 -> D30 Irino 95 mg/day | Not treated |  |
| KDO-0147  01/2016  3 years  Neuroblastoma of the right suprarenal gland  Mts in bones | 1^st^ progression of MIBG score  04/2017  2^nd^ progression  02/2019 | 1^st^ line CTx - Rapid COJEC, 01/2016 - 06/2016  MATIN 06/2016 - 07/2016, 1^st^ PR  HD CTx Bu/Mel + APBSC 10/2016  2^nd^ line CTx - 1 cycle Topo/CPM 01/2017  3^rd^ line CTx - RIST – 2 cycles, 2/2017- 04/2017, PD  4^th^ line CTx - METRO-NB2012, 05/2017 - 12/2018, 2^nd^ PR  Experimental ITx -DC vac course, 02/2019 - ongoing | 26.11.2018  6 years  LS 100 | D60 -> D0  Propranolol 30 mg/day Levothyroxine 37.5 µg/day  D60 -> D47  Celecoxib 200 mg/day | 20.02.2019  PD  LS 100 |  |

**Chemotherapy agents (alphabetically):** Bu – busulphan**;** CPM – cyclophosphamide**;** Doxo – doxorubicin; Eto – etoposide**;** HD MTX – high dose methotrexate; Ifo – ifosfamide; Irino - irinotecan; MATIN – therapeutic MIBG; MIBG - meta-iodobenzylguanidine, radiopharmaceutical; Mel – melphalan; MTX – methotrexate**;** TEM - temsirolimus **;** Topo – topotecan; Treo – treosulphan**;** TZM – temozolomide; VBL – vinblastin**;** VCR – vincristine**.**

**Chemotherapy cycles (alphabetically):** Bu/Mel - high-dose chemotherapy regimen – busulphan and melphalan; Ifo/Eto – chemotherapy cycle – ifosfamide, etoposide; Irino/TZM – chemotherapy cycle – irinotecan, temozolomide**;** IVA – chemotherapy cycle – Ifosfamide, Vincristine, Actinomycin; Topo/CPM- chemotherapy cycle – Topotecan, Cyclofosfamide**;** Treo/Mel – high-dose chemotherapy regimen – treosulfan and melphalan; VAC – chemotherapy cycle - vincristine (1.5 mg/m^2^/day, d1), actinomycin (0.75 mg/m^2^/day, d1, d2), cyclophosphamide (1500mg/m^2^/day, d1);VAI - vincristine (1.5 mg/m^2^/day, d1), actinomycin (0.75 mg/m^2^/day, d1, d2), ifosfamide (3.0 g/m^2^/day, d1, d2); VCR/Irino – chemotherapy cycle – Vincristine, Irinotecan; VDC – chemotherapy cycle – Vincristine (1.5mg/m2/day, d1), Doxorubicin (36 mg/m2/day, d1-2), Cyclophosphamide (1160 mg/m2/day, d1)**;** VIDE – chemotherapy cycle - vincristine (1.5 mg/m^2^/day, d1), ifosfamide (3.0 g/m^2^/day, d1-3), doxorubicin (20 mg/m^2^/day, d1-3), etoposide (150 mg/m^2^/day, d1-3)**;** VTC – chemotherapy cycle – Vincristine (1.5 mg/m^2^/day, d1), Topotecan (0.8 mg/m^2^/day, d1-5), Cyclofosfamide (250 mg/m^2^/day, d1-5).

**Chemotherapy protocols:** AEWS1031 protocol (Ewing sarcoma): combination of vincristine (1.5 mg/m^2^/day, d1 in weeks 1, 2, 5, 6, 9, 10), doxorubcin (37.5 mg/m^2^/day, d1, d2 in weeks 1, 5, 9), cyclophosphamide (1200 mg/m^2^/day, d1 in weeks 1, 5, 9), ifosfamide (1800 mg/m^2^/day, d1-d5 in weeks 3, 7, 11), etoposide (100 mg/m^2^/day, d1-d5 in weeks 3, 7, 11)**.** ANBL 0532 protocol (neuroblastoma): combination of cyclophosphamide (cycle 1,2 - 400 mg/m^2^/day, d1-d5, cycle 4,6 -2100 mg/m^2^/day, d1-d2), topotecan (cycle 1, 2 - 1.2 mg/m^2^/day, d1-d5), cisplatin (cycle 3, 5 - 50 mg/m^2^/day, d1-d4), etoposide (cycle 3, 5 - 200 mg/m^2^/day, d1-d3), doxorubicin (cycle 4, 6 - 25 mg/m^2^/day, d1-d3), vincristine (cycle 4, 6 - 0.67 mg/m^2^ or 0.022 mg/kg/day, d1-d3)**.** AOST 0331 protocol (osteosarcoma): combination of cisplatin (60mg/m^2^/day, d1-d2), doxorubicin (37.5mg/m^2^/day, d1-d2), methotrexate (12g/m^2^/day, d1), cycle Doxo/Cisplatin – weeks 1, 6, 12, 17, cycle Doxo – weeks 22, 26, cycle methotrexate – weeks 4, 5, 9, 10, 15, 16, 20, 21, 24, 25, 28, 29. AOST 1321 - protocol (osteosarcoma): repeated dose of Denosumab = RANKL inhibitor, cycle 1: denosumab 120mg s.c. d1, d8, d15, cycle 2-26: denosumab 120mg s.c. d1. ARTST 08P1 protocol (rhabdomyosarcoma): combination of vincristine (1.5 mg/m^2^/day, cycle 1 – d1, d8, d15; cycles 2, 3, 5, 7 – d1, d8; cycle 9 – d1, d8, d15; cycle 10, 12, 14 – d1, d8; cycles 15, 16, 18 – d1; cycle 17 - d1, d8, d15; cycles 19, 20 – d1, d8), irinotecan (50 mg/m^2^/day, cycles 1, 2, 9, 10, 19, 20 – d1-5), ifosfamide (1800 mg/m^2^/day; cycles 4, 6, 8, 11, 13 – d1-5), etoposide (100 mg/m^2^/day; cycles 4, 6, 8, 11, 13 – d1-5), doxorubicin (37.5 mg/m^2^/day; cycles 3, 5, 7, 12, 14 – d1, 2), cyclophosphamide (1200 mg/m^2^/day; cycles 3, 5, 7, 12, 14-18 – d1), actinomycine (0.045 mg/kg/day; cycles 15-18 – d1). ARST 0921 protocol (refractery or relapsed rhabdomyosarcoma): *regimen A* - 12 cycles vinorelbine (25 mg/m^2^/day d1, d8), cyclophosphamide (1200 mg/m^2^/day, d1), bevacizumab (15 mg/kg/day, d1); *regimen B -* 12 cycles vinorelbine (25 mg/m^2^/day, d1, d8), cyclophosphamide (1200 mg/m^2^/day, d1), temsirolimus (15 mg/m^2^/day d1, d8, d15). ARST 1321 protocol (non-rhabdomyosarcoma soft tissue sarcomas**)**: cycle of ifosfamide (2500 mg/m^2^/day, 3 doses), doxorubicin (37.5 mg/m^2^/day, 2 doses ) every 3 weeks, pazopanib 350 mg/m^2^ daily in weeks 1 – 25. EpSSG RMS 2005 protocol (rhabdomyosarcoma): combination of IVA – ifosfamide (3 g/m^2^/day, d1-2), vincristine (1.5 mg/m^2^ on d1 in weeks 1-7), actinomycin (1.5 mg/m^2^, d1), doxorubicin (30 mg/m^2^, d1-2 for courses 1-4); courses A,B,C,D – combination of cycles IVA and IVA doxorubicin. Euro Ewing 99 protocol (Ewing sarcoma): combination of cycles VIDE, VAI, VAC according to subgroup. Euro Ewing 2008 protocol (Ewing sarcoma): combination of cycles VIDE, VAI, VAC according to subgroup. Rapid Cojec protocol (neuroblastoma): Three different courses are given every 10 days. Course A starts on days 0 and 40, course B on days 10, 30, 50 and 70 and course C on days 20 and 60, *ourse A* - vincristine (1.5 mg/m² , maxium dose 2 mg), carboplatin (750 mg/m²), etoposide (2 x 175mg/m²), *course B* - vincristine (1.5 mg/m², maximum dose 2 mg), cisplatin (80 mg/m²/ctn over 24 hours), *course C* - vincristine (1.5 mg/m², maximum dose 2 mg), etoposide (2 x 175mg/m²), cyclophosphamide (2 x 1050 mg/m²). rEECur protocol (relapsed soft tissue sarcoma): Cycles topotecan/cyclophosphamide (topotecan 0.75 mg/m^2^/day, d1-5; cyclophosphamide 250 mg/m^2^/day, d1-5), cycles irinotecan/temozolomide (irinotecan 50 mg/m^2^/day, d1-5; temozolomide 100 mg/m^2^/day, d1-5). RIST protocol (Neuroblastoma): Combination of rapamycin (3 mg/m^2^ on d1 and 1mg/m^2^, d2-4), dasatinib (300 mg/m^2^/day, d1-4), irinotecan (50 mg/m^2^ i.v.), temozolomide (150 mg/m^2^ p.o./day, d1-5).

**Long term metronomic treatment:** COMBAT III: Year I - celecoxib p.o. (400 mg/m^2^/day, d1-78), etoposide p.o. (25 mg/m^2^/day, d1-35), temozolomide p.o. (30 mg/m^2^/day, d36–77), fenofibrate p.o. (100 mg/m^2^/day, d1-78), ergocalciferol (1500U/m^2^/day, d1-78), bevacizumab (10mg/kg/day, d1, d15, d29, d43, d57, d71); Year II - celecoxib p.o. (400 mg/m^2^/day, d1-78), etoposide p.o. (25 mg/m^2^/day, d1-35), cis-retinoic acid p.o. (100 mg/m^2^/day, d1-14, d29-42, d57-70), fenofibrate p.o. (100 mg/m^2^/day, d1-78), ergocalciferol (1500U/m^2^/day, d1-78). Year III - celecoxib p.o. (400 mg/m^2^/day, d1-78), cis-retinoic acid p.o. (100 mg/m^2^/day, d1-14, d29-42, d57-70), fenofibrate p.o. (100 mg/m^2^/day, d1-78), ergocalciferol (1500U/m^2^/day, d1-78). METRO-NBL2012: Metronomic treatment (neuroblastoma): Treatment consists of eight alternating 28-day-cycles of PCCVE and PCCV followed by five cycles PCCV resulting in a total of 13 cycles (364 days of treatment) PCCVE: propranolol cycle 1 ( 0.5 mg/kg/day p.o. d1, 1 mg/kg/day p.o. d2, 2 mg/kg/day p.o. d3-28), all further cycles (2 mg/kg/day p.o., maximum total daily dose 120 mg, divided in 2 doses per day, d1–28), celecoxib (400 mg/m2xd p.o., maximum total daily dose 800 mg, divided in 2 doses per day. d1-28), cyclophosphamide (loading dose 500 mg/m^2^ i.v., d1, all other cycles 25 mg/m^2^/day p.o., maximum total daily dose 50 mg, d1-28); vinblastine (3 mg/m^2^/day i.v., maximum total daily dose 6 mg, d1, d15, every two weeks), etoposide (25 mg/m^2^/day p.o., maximum total daily dose 50 mg, d1-21). PCCV: propranolol (2 mg/kg/day p.o., maximum total daily dose 120 mg, divided in 2 doses per day, d1–28), celecoxib (400 mg/m^2^/day p.o., maximum total daily dose 800 mg, divided in 2 doses per day, d1-28).

**Other abbreaviations (alphabetically):** Ami – amikacin**;** AmphoB – amphotericin B**;**  AMX/CLA – amoxicillin-clavulanate; APBSC – Autologous Peripheral Blood Stem Cell Transplantation**;** bilat – bilateral or bilaterally**;** Ca-folinate – calcium folinate; Cipro – ciprofloxacin; ctn – continually**;** CR – Complete Response**;** CTx – chemotherapy**;** d – day (in chemotherapeutic protocols)**;** D – day (in treatment prior to mononuclear harvest)**;** DC vac – dendritic cell-based vaccine**;** Dg – diagnosis**;** DOD – died of disease**;** Dinutxmb – dinutuximab; EWS/FLI-1 - a chromosomal 11;22 translocation**;** Gy – gray (unit)**;** ITx – immunotherapy**;** HD CTx – high-dose chemotherapy**;** IRS – intergroup rhabdomyosarcoma study**;** KS – Karnofsky score**;** LMWH – low molecular weight heparin**;** LS – Lansky score**;** M – month**;** mts – metastasis / metastases / metastatic**;** Negat – negative**;** Nmyc – N-myc oncoprotein**;** p.o. – per os**;** Pip/Tazo - Piperacillin/ tazobactam**;** PD – Progressive Disease; Posit – positive**;** PR – Partial Response**;** PS – performance status (Lansky/Karnofsky)**;** RTx – radiotherapy**;** SD – Stable Disease**;** Sub – subject**;** TEP – total endoprosthesis**;** Thx – therapy**;** TMP/SMT – trimethoprim / sulphamethoxazole**;** Y – year**;** Yrs – years
